# Association of Early Physician Follow-up With Readmission Among Patients Hospitalized for Acute Myocardial Infarction, Congestive Heart Failure, or Chronic Obstructive Pulmonary Disease

**DOI:** 10.1001/jamanetworkopen.2022.22056

**Published:** 2022-07-12

**Authors:** Farah E. Saxena, Arlene S. Bierman, Richard H. Glazier, Xuesong Wang, Jun Guan, Douglas S. Lee, Therese A. Stukel

**Affiliations:** 1 Canadian Partnership Against Cancer, Toronto, Canada; 2 ICES, Toronto, Ontario, Canada; 3 Center for Evidence and Practice Improvement, Agency for Healthcare Research and Quality, Rockville, Maryland; 4 Department of Family and Community Medicine, Faculty of Medicine, University of Toronto, Toronto, Ontario, Canada; 5 Institute of Health Policy, Management and Evaluation, University of Toronto, Toronto, Ontario, Canada; 6 Department of Family and Community Medicine, St Michael's Hospital, Toronto, Ontario, Canada; 7 Ted Rogers Centre for Heart Research at the Peter Munk Cardiac Centre, Toronto, Ontario, Canada; 8 Sunnybrook Research Institute, Sunnybrook Health Sciences Centre, Toronto, Ontario, Canada

## Abstract

**Importance:**

A better understanding of the association between early physician follow-up after discharge and adverse outcomes among hospitalized patients may inform interventions aimed at reducing readmission for common chronic conditions.

**Objective:**

To assess whether hospitalized patients with early physician follow-up after discharge had lower rates of overall and condition-specific readmissions within 30 days and 90 days of discharge.

**Design, Setting, and Participants:**

This cohort study was conducted among Ontario, Canada, adults with first admission for acute myocardial infarction (AMI), congestive heart failure (CHF), or chronic obstructive pulmonary disease (COPD) during 2005 to 2019. The exposure was follow-up visit with a primary care physician or relevant specialist within 7 days of discharge. Cox proportional hazards models were used to compare patients with vs without early follow-up, adjusting for sociodemographic factors and comorbidities, weighting by propensity score–based overlap weights. Data were analyzed from January through July 2021.

**Main Outcomes and Measures:**

Primary outcomes were 30-day and 90-day readmissions, cardiac readmissions (readmission for AMI, CHF, or angina) for patients with cardiac conditions, and COPD-related readmissions for patients with COPD. Mortality at 30 days and 90 days was a secondary outcome. All percentages reported in Results are unweighted.

**Results:**

The study cohort comprised 450 746 patients, including 198 854 patients with AMI, 133 058 patients with CHF, and 118 834 patients with COPD; the median (IQR) age was 66 (56-77) years for AMI, 78 (68-85) years for CHF, and 73 (64-81) years for COPD, and there were 64 339 (32.35%) women, 62 575 (47.03%) women, and 59 179 (49.80%) women, respectively. There were 91 182 patients (45.85%), 56 491 patients (42.46%), and 40 159 patients (33.79%), respectively, who received an early follow-up visit. Overall, patients with early follow-up had higher rates of collaborative care (eg, CHF: 20 931 patients [37.85%] vs 11 101 of 76 567 patients [14.85%]) and visits to a specialist within 30 days (eg, CHF: 25 797 patients [45.67%] vs 20 548 patients [26.84%]). Those with early follow-up had lower 90-day readmission rates among patients with CHF (15 934 patients [28.21%] vs 23 121 patients [30.20%]; adjusted hazard ratio [aHR], 0.98; 95% CI, 0.96-0.99) and among those with COPD (8784 patients [21.87%] vs 18 097 of 78 675 patients [23.00%]; aHR, 0.95; 95% CI, 0.93-0.98). Among patients with COPD, those with early follow-up had lower 90-day COPD-related readmission rates (4015 patients [10.00%] vs 8449 patients [10.74%]; aHR, 0.93; 95% CI, 0.89-0.96), and among patients with CHF, those with early follow-up had lower 90-day mortality rates (4044 patients [7.16%] vs 6281 patients [8.20%]; aHR, 0.93; 95% CI, 0.90-0.97). There were no significant benefits at 30 days or for patients with AMI.

**Conclusions and Relevance:**

These findings suggest that early follow-up in conjunction with a comprehensive transitional care strategy for hospitalized patients with medically complex conditions coupled with ongoing effective chronic disease management may be associated with reduced 90-day readmissions.

## Key Points

**Question:**

Is early physician follow-up after discharge associated with 30- and 90-day readmission rates among patients hospitalized with acute myocardial infarction (AMI), congestive heart failure (CHF), or chronic obstructive pulmonary disease (COPD) in Canada?

**Findings:**

In this retrospective cohort study of 450 746 patients with AMI, CHF, or COPD, among individuals with CHF or COPD, those who had early follow-up had lower rates of unplanned readmissions at 90 days. Among patients with COPD, those with early follow-up had lower rates of COPD-related readmissions at 90 days, and among patients with CHF, those with early follow-up had lower 90-day mortality.

**Meaning:**

These findings suggest that early follow-up in conjunction with a comprehensive transitional care strategy for hospitalized patients with medically complex conditions coupled with ongoing effective chronic disease management may be associated with reduced 90-day readmissions.

## Teaser

This cohort study investigates the association of early physician follow-up with readmission among patients hospitalized with acute myocardial infarction, congestive heart failure, or chronic obstructive pulmonary disease.

## Introduction

Unplanned readmissions among hospitalized patients are common and pose a significant burden on patients, families, and the health care system. In Canada, approximately 9.4% of patients are readmitted to the hospital within 30 days of discharge, with the highest readmission rates among patients with congestive heart failure (CHF) and chronic obstructive pulmonary disease (COPD).^1^ While most patients see a physician within 1 month of discharge, less than half of patients with CHF or COPD visit a physician within 7 days of discharge.^1,2,3,4,5^ In the US, similarly high rates of readmission are seen and approximately half of patients visit a physician between discharge and readmission.^6,7,8^

The days immediately after hospital discharge are a period of increased risk for patients. Because of stressors and deconditioning occurring during hospitalization and lingering illness effects, optimal transitions between hospital and ambulatory sectors are key to improving health and reducing readmissions.^9,10^ An early study^11^ found that early physician follow-up among patients with CHF after discharge was associated with reduced 30-day readmissions, leading to early physician follow-up becoming a standard of care.^2,12,13,14,15,16,17^ Early follow-up is an opportunity for clinicians to assess the patient’s condition, comorbidities, medications, and therapies; clarify discharge instructions; and develop a plan if symptoms persist.^2,18^

Good hospital-community transitions may be associated with decreased medication errors, adverse drug events, and readmissions.^19,20^ There have been attempts to standardize the discharge process through medication reconciliation, patient education, communication between hospital and community clinicians, and creation of quality standards^17,19,20,21^ Other efforts have focused on building integrated health care systems in Ontario and the US for treating patients with high levels of need by reorganizing health delivery and payment systems to incentivize coordinated care across hospitals and clinicians, engagement of multidisciplinary teams of primary care and specialists across sectors, and promotion of collective accountability.^22,23,24,25,26^

Our objectives were to assess whether hospitalized patients in Ontario with acute myocardial infarction (AMI), CHF, or COPD who had physician follow-up within 7 days after discharge had lower rates of readmission at 30 and 90 days given that these patients have medically complex conditions, often have unrelated comorbidities, and may be cared for by multiple physicians. We reasoned that 30-day readmissions likely reflect transitional care while 90-day readmissions capture ongoing chronic disease management, although both are a necessary part of care for patients with medically complex conditions.

## Methods

This cohort study used data from ICES, which is a prescribed entity under Ontario’s Personal Health Information Protection Act (PHIPA). Section 45 of PHIPA authorizes ICES to collect personal health information without consent for the purpose of analysis or compiling statistical information with respect to management, evaluation, or monitoring of the allocation of resources to or planning for all or part of the health system. Projects that use data collected by ICES under section 45 of PHIPA and use no other data are exempt from research ethics board review. Use of the data in this project is authorized under section 45 and approved by the ICES Privacy and Legal Office. This report follows the Strengthening the Reporting of Observational Studies in Epidemiology (STROBE) reporting guideline for observational studies.

### Study Cohorts

We undertook a retrospective cohort study of Ontario residents hospitalized with first (ie, index) admission for AMI, CHF, or COPD to an Ontario acute care hospital between April 1, 2005, and December 15, 2019, restricting the population to adults aged 20 to 105 years. We excluded patients who died before discharge and patients who were admitted from or discharged to long-term care, complex continuing care, or rehabilitation because our study context was integration between hospital and community sectors. To reliably assess comorbidities, we excluded patients who were ineligible for Ontario health care during the prior 3 years. We created an index episode of care beginning at initial admission and ending at final discharge, incorporating transfers. We lastly excluded patients who died or were readmitted for any cause within 7 days of discharge among those who survived to discharge but were otherwise eligible (16 223 of 264 457 patients with AMI [6.13%], 17 581 of 256 840 patients with CHF [6.85%], and 16 851 of 276 243 patients with COPD [6.10%]) to allow a window of opportunity to be seen by a physician (Figure).

**Figure.**
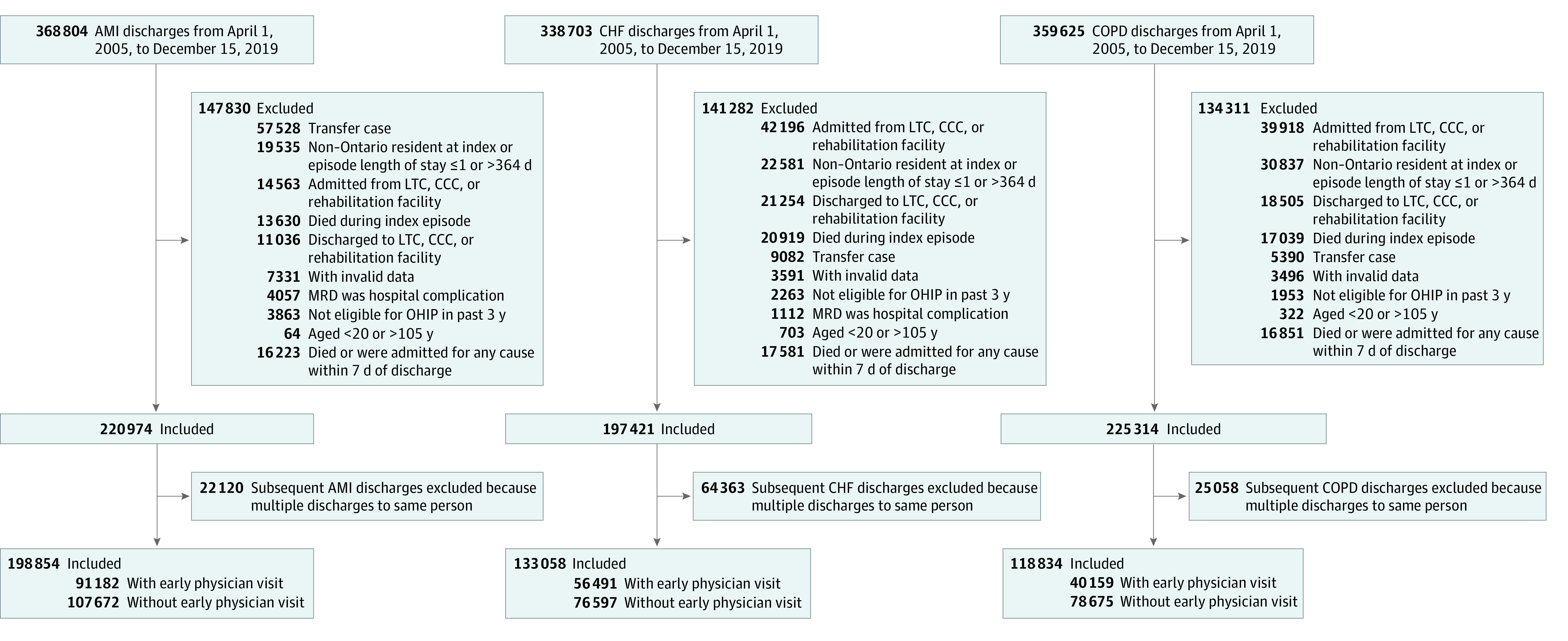
Patient Flowchart Flowchart presents inclusion and exclusion criteria for 3 study cohorts. AMI indicates acute myocardial infarction; CCC, complex continuing care; CHF, congestive heart failure; COPD, chronic obstructive pulmonary disease; LTC, long-term care; MRD, most responsible diagnosis; OHIP, Ontario Health Insurance Plan.

### Exposure

The exposure was an ambulatory visit or telephone call with a primary care (PC) physician (general practitioner or family physician) or relevant specialist within 7 days of discharge. Relevant specialists were cardiologists for AMI and CHF, respirologists for COPD, and general internal medicine specialists for all cohorts.

### Patient Follow-up and Outcomes

Patients were followed up for readmissions and mortality for 90 days after index episode discharge date. Primary outcomes were 30-day and 90-day unplanned all-cause and condition-specific readmission after index admission discharge. Cardiac readmission was defined as readmission for AMI, CHF, or angina. For COPD, we reported readmission for COPD or a COPD-related condition. Mortality was a secondary outcome. Planned subgroup analyses assessed whether findings differed by sex, recency (2005-2012 vs 2013-2019), or length of index hospital stay (LOS; 2 days, 3-7 days, or >7 days) because readmission risk is associated with LOS.^27^ In post hoc analyses, we restricted analyses to frail elderly adults (ages ≥75 years), low-income groups (lowest 3 income quintiles), patients with any comorbidity, and rural patients to investigate whether early physician follow-up had a differential association among these subgroups with increased risks.

### Process Measures

We report visit with a PC physician or relevant specialist within 14 and 30 days; collaborative care as a visit with a PC physician and relevant specialist within 30 days^28,29^; visit with relevant specialist within 30 days; medication reconciliation within 30 days of discharge (eTable 1 in the Supplement); home care services within 30 days of discharge; and prescription of statins, β-blockers, and angiotensin-converting enzyme inhibitors or angiotensin II reception blockers (AMI, CHF), spironolactone (CHF), inhaled and oral corticosteroids, respiratory antibiotics, and long-acting bronchodilators (COPD) for patients aged 65 years or older within 90 days. For patients with CHF, we report presence of left ventricular ejection fraction testing during admission or within 90 days, stress or exercise testing or nuclear perfusion imaging, and permanent pacemaker implantation or implantable cardioverter defibrillator use within 90 days. All percentages presented in Results are unweighted.

### Data Sources

Patient records were linked using unique encoded identifiers and analyzed at ICES. ICES is an independent, nonprofit research institute whose legal status under Ontario’s health information privacy law allows it to collect and analyze health care and demographic data without consent for health system evaluation and improvement. Multiple Ontario health administrative databases were used containing information on all publicly insured, medically necessary hospital and physician services. These comprise the Discharge Abstract Database (DAD) for hospital admissions, procedures, and transfers, which includes the most responsible diagnosis for LOS, secondary diagnosis codes, comorbidities present upon admission, complications occurring during the hospital stay, and attending physician identifier; Ontario Health Insurance Plan for physician billings, which includes diagnosis codes and procedures and location of visit; Ontario Drug Benefit for medication reconciliation and outpatient drug prescriptions for individuals older than age 65 years; and Registered Persons Database for patient demographic information and deaths. PC models were grouped as family health teams (FHT) (interprofessional health care teams), non-FHT models (blended fee-for-service or blended capitation models), or no usual PC physician.^30^ The Ontario Home Care Database was used to record home care visits.

Cause of admission was determined using the most responsible diagnosis, excluding complications occurring during the index hospital episode. Comorbidities were identified using secondary DAD diagnosis fields. Previous study^31^ results suggest the validity and reliability of Ontario’s health administrative data. *International Statistical Classification of Diseases and Related Health Problems, Tenth Revision Canada* (*ICD-10-CA*) diagnosis codes and Canadian Classification of Interventions procedure codes are reported in eTable 2 in the Supplement. Neighborhood income was derived from Statistics Canada 2016 census estimates. Patient residence was measured using the Rurality Index of Ontario (RIO), which accounts for population size and travel time, categorized as urban (RIO < 10) and nonurban (RIO ≥ 10).^32^

### Statistical Analysis

We used propensity score (PS)–based overlap weights to create cohorts that were balanced across baseline characteristics.^33,34^ PS, defined as the probability of receiving vs not receiving an early follow-up visit, was estimated separately for each cohort using multivariable random-effects logistic regression and included covariates associated with receipt of an early follow-up visit.^35,36^ Covariates included age group (20-45, 46-64, 65-74, 75-84, and ≥85 years), sex, condition-specific comorbidities at admission (eTables 3-5 in the Supplement),^29,37,38^ urban residence, neighborhood income quintile, ambulatory visit to a PC physician in the previous year, usual PC physician belonging to an FHT (yes, no, or no usual PC physician),^30^ admission for the same condition in the previous 5 years, and individual Charlson comorbidities in the previous 5 years. PS models for AMI and CHF also included history of CHF or angina in the previous 5 years (AMI) and history of AMI or angina in the previous 5 years (CHF). The PS model for COPD also included the presence of another chronic respiratory disease at admission (eTable 5 in the Supplement). Each cohort was analyzed separately. Hospital random effects were included in the PS to better balance patients discharged from the same hospital given that they were treated similarly in terms of discharge processes.^35^

Each patient was then weighted according to the patient’s overlap weight, defined as the probability of being assigned to the opposite exposure group based on PS.^33^ Overlap weights give larger weights to patients who have a high probability of receiving either exposure, targeting patients who have the greatest overlap in observed covariates and are therefore most in equipoise and downweighting patients in the extremes of the distribution, the reverse of inverse probability treatment weights.^33,34^ Overlap weights achieve perfect balance for covariates included in the PS and produce the smallest standard errors among all balancing-weight approaches.^33,39^

We used Cox proportional hazard regression models, weighting by overlap weights, to estimate adjusted hazard ratios (aHRs) between patients who did vs did not receive an early follow-up visit and included a robust variance estimator to account for weighting.^33,36,40^ We used cause-specific competing risk models to analyze readmissions, treating mortality as a competing risk.^41^ We controlled for covariates included in the propensity model, as well as year of discharge, discharge physician specialty, cardiac revascularization (coronary artery bypass graft surgery or percutaneous coronary intervention) during index admission or in the previous 5 years (AMI), and receipt of implantable cardioverter defibrillator or permanent pacemaker in the previous 3 years (CHF).^29,38^ Statistical tests were 2-sided and performed at the 5% level of significance. Analyses were performed using SAS Enterprise Guide version 7.15 on SAS statistical software version 9.4.5 (SAS Institute).^42^ Data were analyzed from January through July 2021.

## Results

A total of 450 746 patients, including 198 854 patients with AMI, 133 058 patients with CHF, and 118 834 patients with COPD, were eligible for inclusion in the study (Figure). The median (IQR) age was 66 (56-77) years for AMI, 78 (68-85) years for CHF, and 73 (64-81) years for COPD, with 64 339 (32.35%) women, 62 575 (47.03%) women, and 59 179 (49.80%) women, respectively. Of these patients, 91 182 patients (45.85%), 56 491 patients (42.46%), and 40 159 patients (33.79%), respectively, received a 7-day physician follow-up visit, and these early physician visit rates did not change over time (eTable 6 in the Supplement); there were 107 672 patients (54.15%), 76 567 patients (57.54%), and 78 675 patients (66.20%) without an early follow-up visit, respectively. Before weighting, patients with an early follow-up visit tended to be urban dwellers (eg, CHF: 39 341 patients [69.64%] vs 47 932 patients [62.60%]) and more likely to have visited a PC physician in the previous year (eg, CHF: 53 076 patients [93.95%] vs 66 888 patients [87.36%]) compared with patients without early follow-up. Patients with AMI who had an early visit were younger, more likely to be discharged from a high-volume hospital and undergo cardiac revascularization during index admission, and less likely to be admitted for a cardiac condition within the prior 5 years (Table 1). Patients with CHF who had an early visit were more likely to have arrhythmia and a cardiologist as discharge physician. After weighting, there were no differences between exposure groups in sociodemographic characteristics, comorbidities, medical history, or access to primary care (Table 1). Most patients who had an early visit with a PC physician saw their usual provider of primary care (eg, CHF: 48 817 of 56 491 patients [86.42%]).

**Table 1.** Selected Baseline Patient, Physician, and Hospital Characteristics

Characteristic	Before weighting			After weighting^a^		
	Patients, No. (%)		Standardized difference	Patients, %		Standardized difference
	7-d Follow-up	No 7-d follow-up		7-d Follow-up	No 7-d follow-up	
**AMI**						
Patients, No.	91 182	107 672	NA	NA	NA	NA
Patient characteristic						
Age ≥65 y	45 433 (49.83)	59 870 (55.60)	0.11	52.60	52.60	0
Women	27 564 (30.23)	36 775 (34.15)	0.08	32	32	0
High income^b^	34 507 (37.84)	38 041 (35.33)	0.05	36.89	36.89	0
Urban residence (RIO score <10)	59 612 (65.38)	62 243 (57.81)	0.16	62.26	62.26	0
LOS, mean (SD), d	6.38 (6.53)	6.85 (7.55)	0.06	6.49 (4.84)	6.64 (4.82)	0.02
Comorbidity						
Shock	743 (0.81)	814 (0.76)	0.01	0.78	0.78	0
CHF	8482 (9.30)	11 620 (10.79)	0.05	9.96	9.96	0
Cancer	1472 (1.61)	2206 (2.05)	0.03	1.83	1.83	0
Cerebrovascular disease	659 (0.72)	966 (0.90)	0.02	0.81	0.81	0
Diabetes with complication	21 602 (23.69)	26 870 (24.96)	0.03	24.23	24.23	0
Cardiac dysrhythmia	8998 (9.87)	10 475 (9.73)	0	9.85	9.85	0
Acute kidney failure	2592 (2.84)	3599 (3.34)	0.03	3.09	3.09	0
Chronic kidney failure	2575 (2.82)	4366 (4.05)	0.07	3.30	3.30	0
Pulmonary edema	248 (0.27)	365 (0.34)	0.01	0.30	0.30	0
Ambulatory visit to PC in previous 1 y	78 815 (86.44)	83 912 (77.93)	0.22	83.36	83.36	0
Admission for AMI in previous 5 y	3974 (4.36)	6289 (5.84)	0.07	4.93	4.93	0
Admission for CHF or angina in previous 5 y	7592 (8.33)	11 871 (11.03)	0.09	49.42	9.42	0
Cardiac revascularization during index admission	61 384 (67.32)	65 275 (60.62)	0.14	65.57	62.99	0.05
Cardiac revascularization in previous 5 y	4310 (4.73)	5831 (5.42)	0.03	5.00	5.08	0
Physician characteristic						
Usual PC provider belongs to FHT PC model	21 769 (23.87)	28 709 (26.66)	0.06	25.34	25.34	0
Discharge physician specialty						
Cardiology	47 091 (51.65)	54 080 (50.23)	0.03	51.34	51.07	0
General internal medicine	23 776 (26.08)	27 005 (25.08)	0.02	25.43	26.22	0.01
FP or GP	10 521 (11.54)	16 485 (15.31)	0.11	12.52	13.52	0.03
Hospital characteristic						
Index hospital volume^c^						
High, community	35 333 (38.75)	36 267 (33.68)	0.10	36.42	36.36	0
High, teaching	18 642 (20.44)	23 058 (21.42)	0.02	20.99	21.00	0
**CHF**						
Patients, No.	56 491	76 567	NA	NA	NA	NA
Patient characteristic						
Age ≥65 y	46 344 (82.04)	63 006 (82.29)	0.01	82.21	82.21	0
Women	25 239 (44.68)	37 336 (48.76)	0.08	46.47	46.47	0
High income^b^	19 352 (34.26)	24 459 (31.94)	0.05	33.34	33.34	0
Urban residence (RIO <10)	39 341 (69.64)	47 932 (62.60)	0.15	66.89	66.89	0
LOS, mean (SD), d	7.68 (7.81)	8.33 (8.79)	0.08	7.68 (5.81)	8.29 (5.53)	0.07
Comorbidity						
Ischemic heart disease	13 545 (23.98)	17 695 (23.11)	0.02	23.61	23.61	0
Shock	170 (0.30)	212 (0.28)	0	0.29	0.29	0
Peripheral vascular disease	1347 (2.38)	1925 (2.51)	0.01	2.45	2.45	0
Arrhythmia	20 542 (36.36)	23 084 (30.15)	0.13	33.52	33.52	0
Cerebrovascular disease	582 (1.03)	797 (1.04)	0	1.03	1.03	0
Hypertension	20 655 (36.56)	27 130 (35.43)	0.02	36.10	36.10	0
COPD	7671 (13.58)	11 927 (15.58)	0.05	14.40	14.40	0
Dementia	1045 (1.85)	1976 (2.58)	0.05	2.11	2.11	0
Acute kidney failure	6533 (11.56)	8405 (10.98)	0.02	11.37	11.37	0
Chronic renal failure	1586 (2.81)	2604 (3.40)	0.03	3.05	3.05	0
Diabetes	21 841 (38.66)	30 110 (39.33)	0.01	39.03	39.03	0
Nonmetastatic cancer	1956 (3.46)	2724 (3.56)	0	3.54	3.54	0
Metastatic cancer	441 (0.78)	614 (0.80)	0	0.80	0.80	0
Moderate or severe liver disease	146 (0.26)	169 (0.22)	0.01	0.24	0.24	0
Other cardiovascular disease	12 029 (21.29)	14 638 (19.12)	0.05	20.33	20.33	0
Ambulatory visit to PC in previous 1 y	53 076 (93.95)	66 888 (87.36)	0.23	92.12	92.12	0
Admission for CHF in previous 5 y	16 775 (29.69)	25 018 (32.67)	0.06	31.01	31.01	0
Admission for AMI or angina in previous 5 y	11 886 (21.04)	17 395 (22.72)	0.04	21.81	21.81	0
Cardiac revascularization in previous 5 y	6938 (12.28)	8897 (11.62)	0.02	12.31	11.61	0.02
Implantable cardiac defibrillator in previous 3 y	3270 (5.79)	3956 (5.17)	0.03	5.79	5.23	0.02
Permanent pacemaker in previous 3 y	60 (0.11)	76 (0.10)	0	0.11	0.10	0
Physician characteristic						
Usual PC provider belongs to FHT PC model	13 173 (23.32)	19 759 (25.81)	0.06	24.48	24.48	0
Discharge physician specialty						
Cardiology	15 291 (27.07)	16 826 (21.98)	0.11	25.39	24.22	0.02
General internal medicine	21 421 (37.92)	27 010 (35.28)	0.05	37.34	37.05	0
FP or GP	15 741 (27.86)	26 032 (34.00)	0.13	29.94	30.41	0.01
Hospital characteristic						
Index hospital volume^c^						
High, community	22 637 (40.07)	27 863 (36.39)	0.07	38.76	38.62	0
High, teaching	15 652 (27.71)	19 560 (25.55)	0.05	26.88	26.80	0
**COPD**						
Patients, No.	40 159	78 675	NA	NA	NA	NA
Patient characteristic						
Age ≥65 y	29 662 (73.86)	58 882 (74.84)	0.02	74.29	74.29	0
Women	19 100 (47.56)	40 079 (50.94)	0.06	48.82	48.82	0
High income^b^	12 218 (30.42)	22 355 (28.41)	0.04	29.75	29.75	0
Urban residence (RIO <10)	23 682 (58.97)	41 592 (52.87)	0.12	57.17	57.17	0
LOS, mean (SD), d	6.55 (6.65)	6.96 (7.41)	0.06	6.58 (5.39)	6.94 (4.15)	0.05
Comorbidity						
Ischemic heart disease	4014 (10.00)	7220 (9.18)	0.03	9.70	9.70	0
Shock	65 (0.16)	128 (0.16)	0	0.16	0.16	0
Peripheral vascular disease	701 (1.75)	1372 (1.74)	0	1.74	1.74	0
Arrhythmia	5000 (12.45)	7785 (9.90)	0.08	11.47	11.47	0
Cerebrovascular disease	317 (0.79)	638 (0.81)	0	0.80	0.80	0
Hypertension	9413 (23.44)	17 155 (21.80)	0.04	22.86	22.86	0
Dementia	653 (1.63)	1566 (1.99)	0.03	1.76	1.76	0
CHF	5178 (12.89)	9526 (12.11)	0.02	12.63	12.63	0
Other cardiovascular disease	2010 (5.01)	3724 (4.73)	0.01	4.91	4.91	0
Ambulatory visit to PC in previous 1 y	37 661 (93.78)	67 469 (85.76)	0.27	92.16	92.16	0
Admission for COPD in previous 5 y	13 557 (33.76)	27 957 (35.53)	0.04	34.44	34.44	0
Other chronic respiratory disease	1661 (4.14)	3108 (3.95)	0.01	4.09	4.09	0
Physician characteristic						
Usual PC provider belongs to FHT PC model	10 396 (25.89)	22 674 (28.82)	0.06	26.90	26.90	0
Discharge physician specialty						
Respirology	3847 (9.58)	7376 (9.38)	0.01	9.40	9.92	0.02
General internal medicine	13 642 (33.97)	23 488 (29.85)	0.09	32.93	32.46	0.01
FP or GP	19 657 (48.95)	41 387 (52.61)	0.07	50.15	49.48	0.01
Hospital characteristic						
Index hospital volume^c^						
High, community	19 386 (48.27)	36 222 (46.04)	0.04	47.70	47.57	0
High, teaching	8651 (21.54)	16 991 (21.60)	0	21.79	21.77	0

Abbreviations: AMI, acute myocardial infarction; CHF, congestive heart failure; COPD, chronic obstructive pulmonary disease; FHT, family health team; FP, family physician; GP, general practitioner; LOS, length of stay; NA, not applicable; PC, primary care; RIO, Rurality Index of Ontario.

^a^ Weighting is by propensity-based overlap weights.

^b^ Neighborhood income was derived from Statistics Canada 2016 census estimates. High income refers to the 2 highest income quintiles.

^c^ High patient volume was defined as more than 300 patients with AMI per year for AMI, more than 250 patients with CHF per year for CHF, and more than 200 patients with COPD per year for COPD.

Patients who received a 7-day physician follow-up visit were more than 2-fold as likely to receive collaborative care (ie, visit both a PC physician and relevant specialist) within 30 days (eg, CHF: 20 931 patients [37.85%] vs 11 101 patients [14.85%]) and up to 43% more likely to see a relevant specialist within 30 days (eg, CHF: 25 797 [45.67%] vs 20 548 patients [26.84%]) compared with patients without a 7-day follow-up. They were more likely to receive medication reconciliation (eg, CHF: 7066 patients [15.25%] vs 8836 patients [14.02%]) and be prescribed evidence-based medications within 90 days (eg, CHF: 29 319 patients [63.26%] vs 37 947 patients [60.23%]) (Table 2). Patients with CHF who had a 7-day follow-up were more likely to receive a left ventricular ejection fraction assessment (38 899 patients [74.17%] vs 48 628 patients [69.19%]) and noninvasive testing for ischemia (5482 patients [10.45%] vs 5635 patients [8.02%]). Of patients who did not see a physician within 7 days, approximately half saw a physician within 14 days (eg, CHF: 31 061 patients [40.57%]) and 75% within 30 days (eg, CHF: 53 595 patients [70.00%]). All adverse event rates were higher for patients aged 75 years and older, increased with LOS for all cohorts, and were higher for female than male patients with AMI (eTable 7 in the Supplement).

**Table 2.** Selected Patient Therapies and Procedures

Therapy or procedure	Patients before weighting, No. (%)		Patients after weighting^a^	
	7-d Follow-up	No 7-d follow-up	7-d Follow-up	No 7-d follow-up
**AMI**				
Patients, No.	91 182	107 672	NA	NA
Postdischarge care				
Visit with PC physician or relevant specialist^b^				
Within 14 d	91 182 (100.00)	49 066 (45.57)	100	47.78
Within 30 d	91 182 (100.00)	80 076 (74.37)	100	76.61
Collaborative care within 30 d^c^	29 071 (32.08)	15 571 (14.58)	30.98	16.05
Visit with relevant specialist within 30 d^b^	33 176 (36.38)	25 644 (23.82)	35.78	25.13
Medication reconciliation within 30 d	9615 (21.16)	11 437 (19.10)	21.03	19.35
Home care service within 30 d	16 301 (17.88)	24 491 (22.75)	19.22	20.87
Discharge drug prescription among patients aged ≥65 y				
Statin within 90 d	41 706 (91.80)	53 626 (89.57)	91.35	90.35
β-blocker within 90 d	36 300 (79.90)	47 023 (78.54)	79.85	78.91
ACEI or ARB within 90 d	36 368 (80.05)	46 472 (77.62)	79.41	78.57
**CHF**				
Patients, No.	56 491	76 567	NA	NA
Postdischarge care				
Visit with PC physician or relevant specialist^b^				
Within 14 d	56 491 (100.00)	31 061 (40.57)	100	42.33
Within 30 d	56 491 (100.00)	53 595 (70.00)	100	72.04
Collaborative care within 30 d^c^	20 931 (37.85)	11 101 (14.85)	36.25	16.48
Visit with relevant specialist within 30 d^b^	25 797 (45.67)	20 548 (26.84)	44.48	28.66
Medication reconciliation within 30 d	7066 (15.25)	8836 (14.02)	15.17	14.11
Home care service within 30 d	27 242 (48.22)	41 554 (54.27)	49.18	53.19
Permanent pacemaker implantation or ICD within 90 d	6126 (11.68)	6251 (8.89)	11.34	9.25
Postdischarge testing				
LVEF assessment during admission or within 90 d	38 899 (74.17)	48 628 (69.19)	73.17	70.88
Stress or exercise testing, nuclear MPI within 90 d	5482 (10.45)	5635 (8.02)	10.18	8.32
Discharge drug prescriptions, among individuals aged ≥65 y				
Statin within 90 d	29 319 (63.26)	37 947 (60.23)	62.92	60.84
β-blocker within 90 d	32 394 (69.90)	42 213 (67.00)	69.51	67.69
ACEI or ARB within 90 d	30 601 (66.03)	40 718 (64.63)	65.65	65.02
Spironolactone within 90 d	11 330 (24.45)	14 180 (22.51)	24.23	22.81
**COPD**				
Patients, No.	40 159	78 675		
Postdischarge care				
Visit with PC physician or relevant specialist^b^				
Within 14 d	40 159 (100.00)	28 559 (36.30)	100.00	38.13
Within 30 d	40 159 (100.00)	50 593 (64.31)	100.00	66.72
Collaborative care within 30 d^c^	7597 (19.25)	5534 (7.15)	18.56	8.05
Visit with relevant specialist within 30 d^b^	9421 (23.46)	10 555 (13.42)	22.93	14.39
Medication reconciliation within 30 d	3057 (10.31)	5721 (9.72)	10.29	9.82
Home care service within 30 d	16 199 (40.34)	35 366 (44.95)	40.84	44.43
Discharge drug prescriptions, among individuals aged ≥65 y				
Long-acting bronchodilator within 90 d	21 453 (72.32)	41 600 (70.65)	72.02	71.22
Inhaled corticosteroid within 90 d	19 222 (64.80)	36 670 (62.28)	64.48	62.82
Oral corticosteroid within 90 d	15 781 (53.20)	29 346 (49.84)	53.03	50.28
Respiratory antibiotic within 90 d	19 737 (66.54)	36 247 (61.56)	66.48	61.50

Abbreviations: ACEI, angiotensin-converting-enzyme inhibitor; AMI, acute myocardial infarction; ARB, angiotensin II reception blocker; CHF, congestive heart failure; COPD, chronic obstructive pulmonary disease; ICD, implantable cardioverter defibrillator; LVEF, left ventricular ejection fraction; MPI, myocardial perfusion imaging; PC, primary care.

^a^ Weighting is by propensity-based overlap weights.

^b^ Relevant specialist was cardiologist or general internal medicine specialist for AMI and CHF and respirologist or general internal medicine specialist for COPD.

^c^ Visit with PC physician and relevant specialist.

Among patients with CHF and COPD, those with 7-day follow-up had fewer readmissions within 90 days (CHF: 15 934 patients [28.21% vs 23 121 patients [30.20%]; aHR, 0.98; 95% CI, 0.96-0.99; number needed to treat [NNT] = 139 patients; COPD: 8784 patients [21.87%] vs 18 097 patients [23.00%]; aHR, 0.95; 95% CI, 0.93-0.98; NNT = 98 patients) compared with those without 7-day follow-up (Table 3). Among patients with COPD, those with 7-day follow-up had fewer readmissions for COPD-related conditions within 90 days of discharge (4015 patients [10.00%] vs 8449 patients [10.74%]; aHR, 0.93; 95% CI, 0.89-0.96; NNT = 135 patients), while among patients with CHF, those with 7-day follow-up had lower mortality rates at 90 days (4044 patients [7.16%] vs 6281 patients [8.20%]; aHR, 0.93; 95% CI, 0.90-0.97; NNT = 208 patients). For patients with COPD, those with 7-day follow-up had lower 30-day readmissions (aHR, 0.97; 95% CI, 0.93-1.00) and COPD-related readmissions (aHR, 0.94; 95% CI, 0.89-1.00), but these differences were not statistically significant (Table 3). There were no significant differences for patients with AMI and no benefits at 30 days for any cohort. Models are reported in eTables 8 through 25 in the Supplement. Findings were generally not different across sex, LOS, or subgroup at increased risk or in recent years (eTable 26 in the Supplement).

**Table 3.** Rates and Adjusted Hazard Ratios of Adverse Events

Outcome	Unadjusted rate				HR (95% CI)	
	Patients before weighting, No. (%)		Patients after weighting, %^a^		Unadjusted	Adjusted after weighting^a^
	7-d Follow-up	No 7-d follow-up	7-d Follow-up	No 7-d follow-up		
**AMI**						
Patients, No.	91 182	107 672	NA	NA	NA	NA
Unplanned readmission within 30 d of discharge	5837 (6.40)	7805 (7.25)	6.71	6.78	0.88 (0.85-0.91)	0.99 (0.95-1.02)
Major cardiac event within 30 d of discharge	2097 (2.30)	2836 (2.63)	3..02	2.99	0.87 (0.82-0.92)	1.02 (0.97-1.08)
Death within 30 d of discharge	548 (0.60)	901 (0.84)	0.68	0.73	0.72 (0.65-0.80)	0.93 (0.83-1.04)
Unplanned readmission within 90 d of discharge	11 795 (12.94)	15 856 (14.73)	13.64	13.69	0.87 (0.85-0.89)	0.99 (0.97-1.02)
Major cardiac event within 90 d of discharge	4412 (4.84)	6050 (5.62)	5.22	5.13	0.86 (0.82-0.89)	1.02 (0.98-1.06)
Death within 90 d of discharge	1701 (1.87)	2781 (2.58)	2.13	2.26	0.72 (0.68-0.76)	0.95 (0.89-1.01)
**CHF**						
Patients, No.	56 491	76 567	NA	NA	NA	NA
Unplanned readmission within 30 d of discharge	7490 (13.26)	10 593 (13.83)	13.54	13.50	0.96 (0.93-0.99)	1.01 (0.98-1.04)
Major cardiac event within 30 d of discharge	3356 (5.94)	4612 (6.02)	6.04	5.91	0.99 (0.94-1.03)	1.02 (0.98-1.07)
Death within 30 d of discharge	1195 (2.12)	1807 (2.36)	2.20	2.26	0.90 (0.83-0.96)	0.98 (0.91-1.05)
Unplanned readmission within 90 d of discharge	15 934 (28.21)	23 121 (30.20)	28.74	29.49	0.93 (0.91-0.94)	0.98 (0.96-0.99)
Major cardiac event within 90 d of discharge	7469 (13.22)	10 453 (13.65)	13.42	13.39	0.96 (0.94-0.99)	1.00 (0.97-1.03)
Death within 90 d of discharge	4044 (7.16)	6281 (8.20)	7.40	7.91	0.87 (0.83-0.90)	0.93 (0.90-0.97)
**COPD**						
Patients, No.	40 159	78 675	NA	NA	NA	NA
Unplanned readmission within 30 d of discharge	4072 (10.14)	8296 (10.54)	10.19	10.54	0.96 (0.93-1.00)	0.97 (0.93-1.00)
Readmission for COPD or COPD-related condition within 30 d of discharge	1718 (4.28)	3581 (4.55)	4.29	4.54	0.94 (0.89-1.00)	0.94 (0.89-1.00)
Death within 30 d of discharge	693 (1.73)	1319 (1.68)	1.75	1.68	1.03 (0.94-1.13)	1.04 (0.95-1.15)
Unplanned readmission within 90 d of discharge	8784 (21.87)	18 097 (23.00)	21.97	22.97	0.95 (0.92-0.97)	0.95 (0.93-0.98)
Readmission for COPD or COPD-related condition within 90 d of discharge	4015 (10.00)	8449 (10.74)	10.02	10.74	0.93 (0.89-0.96)	0.93 (0.89-0.96)
Death within 90 d of discharge	2105 (5.24)	4361 (5.54)	5.31	5.54	0.95 (0.90-1.00)	0.96 (0.91-1.01)

Abbreviations: AMI, acute myocardial infarction; CHF, congestive heart failure; COPD, chronic obstructive pulmonary disease; HR, hazard ratio; NA, not applicable.

^a^ Weighting is by propensity-based overlap weights.

## Discussion

This population-based retrospective cohort study found that early follow-up with a PC physician or relevant specialist was associated with fewer readmissions for patients with CHF or COPD, fewer COPD-related readmissions for patients with COPD, and lower mortality for patients with CHF, all within 90 days of discharge, but that there was no demonstrable benefit at 30 days or for patients with AMI. Less than half of patients discharged from an Ontario hospital after an admission did not see a physician within 7 days, and the percentages did not change over 15 years despite the importance of early follow-up.

This study’s strengths are the large population-based cohort examining outcomes after early physician follow-up across 3 serious conditions. The study was conducted in a single-payer health care system with few financial barriers to care, used rigorous methods, and included comprehensive measures of health care use, outcomes, and performance on factors associated with process.

The finding that there were no associations at 30 days may be associated with study patients being healthier than the overall discharged patient population because we excluded 6.13% of patients with AMI, 6.85% of patients with CHF, and 6.10% of patients with COPD who died or were readmitted within 7 days of discharge. It may take more than 30 days for the occurrence of outcomes associated with interventions begun at the 7-day follow-up visit. Several factors may explain the small effect sizes. Patients in groups with and without 7-day follow-up received high levels of evidence-based care, including medications, home care, and testing, and had high continuity of care with their usual PC physicians, and most patients not seeing a physician within 7 days had a visit within 14 days, which may have attenuated associations. It is also possible that patients may require follow-up earlier than 7 days. Randomized clinical trials are underway to evaluate whether early (72-hour) cardiology follow-up, risk stratification, and systematized outpatient care can reduce readmissions among patients with CHF who present to the emergency department.^43^

Most importantly, early physician visits are only 1 component of a large set of collaborative efforts required for better patient care. In our study, patients with early follow-up also had higher rates of collaborative care, visits with a relevant specialist, guideline-directed medical therapy, and medication reconciliation, and patients with CHF had higher rates of cardiac functional assessment and evaluation for myocardial ischemia, all of which may have been associated with improvement, suggesting that early physician visits may provide the opportunity to develop a plan of care and ongoing chronic disease management. AMI care pathways may also be more standardized and responsive to acute management than those for chronic diseases like CHF and COPD. Systematic review^19,44,45,46^ findings suggest that care including a multilevel discharge planning approach and care coordination in predischarge (discharge planning, medication reconciliation, and scheduling follow-up visits), postdischarge (patient hotlines, timely physician follow-up, home visits, and chronic disease management), and transitional settings may be optimal and associated with reduced readmissions and patient improvement. Meta-analyses^20,47,48^ of randomized clinical trials of patients with CHF found that comprehensive discharge planning with postdischarge support, multidisciplinary heart failure–management clinics, structured telephone support, and nurse home-visiting programs was associated with reduced readmissions and mortality.

Reasons for readmission are multifactorial. In our study, the rate of all-cause readmissions was 2-fold that of condition-specific readmissions, which suggests that these patients may have also had unrelated comorbid conditions. Multimorbidity is associated with increased risk of adverse events and requires a multidisciplinary approach to care.^49^ Guidance and tool kits have been developed to address multiple chronic conditions, including by providing appropriate medication management and care integration, while optimizing quality of life.^49,50^

Other studies^29,51^ have found comparable results. In Ontario, early physician follow-up of patients with CHF after discharge from the emergency department was associated with lower readmission rates and reduced mortality at 90 days, although the decrease in mortality was not statistically significant. Patients with CHF who had US Medicare and were discharged from hospitals with higher rates of physician follow-up within 7 days had lower risk of 30-day readmission,^11,52^ but this was not true among patients with AMI.^18,53^ Among patients with COPD who had US Medicare and had a follow-up physician visit within 30 days of discharge, there was a reduced risk of 30-day readmission.^54^ A randomized clinical trial of patients with CHF across 10 Canadian hospitals^55^ found that after a transitional care intervention, including physician follow-up within 1 week of discharge, the composite outcome of readmission or emergency department visit within 30 days was lower (aHR, 0.93), although this difference was not statistically significant.

### Limitations

This study has several limitations. A lack of clinical data prevented us from understanding the care patients received, including the content and quality of physician visits and the extent of communication between discharge and community physicians or PC physicians and specialists. As in all studies using administrative data, our data on patient clinical presentation were limited to recorded diagnoses. Thus, findings may be biased owing to lack of information on unmeasured risk factors, such as smoking status, disease severity, adherence to recommendations, self-efficacy in disease management, and social risk. Additionally, our findings are limited to Ontario and so may not generalize to other jurisdictions; however, given Ontario’s size and diversity, these findings may be likely to reflect trends in the rest of the country.

## Conclusions

These findings may have implications for performance measurement and practice improvement. Early postdischarge physician visits may be important to maximize the reduction in adverse events associated with treatment for patients with medically complex conditions. However, these visits need to be part of a comprehensive transitional care strategy coupled with ongoing effective chronic disease management encompassing care coordination among multiple sectors of the health care system and providing comprehensive, patient-centered care that addresses coexisting illness. Early physician follow-up and readmission rates did not change in 15 years, despite performance measurement and reporting, suggesting the need to better develop, implement, and evaluate comprehensive models of care that address factors associated with readmission, along with implementation strategies associated with improvement.

## Notes

Supplement. **eTable 1.** Medication Reconciliation: Ontario Drug Benefit Claims for Selected Drug Information Numbers **eTable 2.** Canadian Classification of Interventions Procedure Codes for Cardiac Conditions **eTable 3.** Congestive Heart Failure and Chronic Obstructive Pulmonary Disease Comorbidities at Index Admission **eTable 4.** Additional Congestive Heart Failure Comorbidities at Index Admission **eTable 5.** Additional Chronic Obstructive Pulmonary Disease Comorbidities at Index Admission **eTable 6.** Postdischarge Physician Visits by Fiscal Years (FYs) 2005-2012 and 2013-2018 **eTable 7.** Adverse Outcome Rates Overall and by Sex, Length of Stay, and Subgroup at Increased Risk **eTable 8.** Cox Proportional Hazards Model for Death Within 30 d Among Patients With Acute Myocardial Infarction **eTable 9. **Cox Proportional Hazards Model for All-Cause Readmissions (Death as Competing Risk) Within 30 d Among Individuals With Acute Myocardial Infarction **eTable 10.** Cox Proportional Hazards Model for Major Cardiac Event (Death as Competing Risk) Within 30 d Among Individuals With Acute Myocardial Infarction **eTable 11.** Cox Proportional Hazards Model for Death Within 90 d Among Individuals With Acute Myocardia Infarction **eTable 12.** Cox Proportional Hazards Model for All-Cause Readmissions (Death as Competing Risk) Within 90 d Among Individuals With Acute Myocardial Infarction **eTable 13.** Cox Proportional Hazards Model for Major Cardiac Event (Death as Competing Risk) Within 90 d Among Individuals With Acute Myocardial Infarction **eTable 14.** Cox Proportional Hazards Model for Death Within 30 d Among Individuals With Congestive Heart Failure **eTable 15.** Cox Proportional Hazards Model for All-Cause Readmissions (Death as Competing Risk) Within 30 d Among Individuals With Congestive Heart Failure **eTable 16.** Cox Proportional Hazards Model for Major Cardiac Event (Death as Competing Risk) Within 30 d Among Individuals With Congestive Heart Failure **eTable 17. **Cox Proportional Hazards Model for Death Within 90 d Among Individuals With Congestive Heart Failure **eTable 18. **Cox Proportional Hazards Model for All-Cause Readmissions (Death as Competing Risk) Within 90 d Among Individuals With Congestive Heart Failure **eTable 19. **Cox Proportional Hazards Model for Major Cardiac Event (Death as Competing Risk) Within 90 d Among Individuals With Congestive Heart Failure **eTable 20.** Cox Proportional Hazards Model for Death Within 30 d Among Individuals With Chronic Obstructive Pulmonary Disease **eTable 21. **Cox Proportional Hazards Model for All-Cause Readmissions (Death as Competing Risk) Within 30 d Among Individuals With Chronic Obstructive Pulmonary Disease **eTable 22.** Cox Proportional Hazards Model for Readmission for Chronic Obstructive Pulmonary Disease (COPD) or COPD-Related Condition (Death as Competing Risk) Within 90 d Among Individuals With COPD **eTable 23.** Cox Proportional Hazards Model for Death Within 90 Days Among Individuals With Chronic Obstructive Pulmonary Disease **eTable 24.** Cox Proportional Hazards Model for All-Cause Readmission (Death as Competing Risk) Within 90 d Among Individuals With Chronic Obstructive Pulmonary Disease **eTable 25.** Cox Proportional Hazards Model for Readmission for Chronic Obstructive Pulmonary Disease (COPD) or COPD-Related Conditions (Death as Competing Risk) Within 90 d Among Individuals With COPD **eTable 26.** Adjusted Hazard Ratios With 95% CIs of Adverse Events for Patients Who Received vs Did Not Receive Early Physician Follow-up, Overall, by Sex, Length of Stay Group, and Subgroup at Increased Risk
