# Supplementary material for: Association of Early Physician Follow-up With Readmission Among Patients Hospitalized for Acute Myocardial Infarction, Congestive Heart Failure, or Chronic Obstructive Pulmonary Disease
Source: JAMA Netw Open. 2022 Jul 12;5(7):e2222056. doi: 10.1001/jamanetworkopen.2022.22056 (PMC9277500; doi:10.1001/jamanetworkopen.2022.22056)
Supplement: Supplement. — eTable 1. Medication Reconciliation: Ontario Drug Benefit Claims for Selected Drug Information Numbers eTable 2. Canadian Classification of Interventions Procedure Codes for Cardiac Conditions eTable 3. Congestive Heart Failure and Chronic Obstructive Pulmonary Disease Comorbidities at Index Admission eTable 4. Additional Congestive Heart Failure Comorbidities at Index Admission eTable 5. Additional Chronic Obstructive Pulmonary Disease Comorbidities at Index Admission eTable 6. Postdischarge Physician Visits by Fiscal Years (FYs) 2005-2012 and 2013-2018 eTable 7. Adverse Outcome Rates Overall and by Sex, Length of Stay, and Subgroup at Increased Risk eTable 8. Cox Proportional Hazards Model for Death Within 30 d Among Patients With Acute Myocardial Infarction eTable 9. Cox Proportional Hazards Model for All-Cause Readmissions (Death as Competing Risk) Within 30 d Among Individuals With Acute Myocardial Infarction eTable 10. Cox Proportional Hazards Model for Major Cardiac Event (Death as Competing Risk) Within 30 d Among Individuals With Acute Myocardial Infarction eTable 11. Cox Proportional Hazards Model for Death Within 90 d Among Individuals With Acute Myocardia Infarction eTable 12. Cox Proportional Hazards Model for All-Cause Readmissions (Death as Competing Risk) Within 90 d Among Individuals With Acute Myocardial Infarction eTable 13. Cox Proportional Hazards Model for Major Cardiac Event (Death as Competing Risk) Within 90 d Among Individuals With Acute Myocardial Infarction eTable 14. Cox Proportional Hazards Model for Death Within 30 d Among Individuals With Congestive Heart Failure eTable 15. Cox Proportional Hazards Model for All-Cause Readmissions (Death as Competing Risk) Within 30 d Among Individuals With Congestive Heart Failure eTable 16. Cox Proportional Hazards Model for Major Cardiac Event (Death as Competing Risk) Within 30 d Among Individuals With Congestive Heart Failure eTable 17. Cox Proportional Hazards Model for Death Within 90 d Among I [file jamanetwopen-e2222056-s001.pdf]

Supplemental Online Content

Saxena FE, Bierman AS, Glazier RH, et al. Association of early physician follow-up with readmission among patients hospitalized for acute myocardial infarction, congestive heart failure, or chronic obstructive pulmonary disease. *JAMA Netw Open*. 2022;5(7):e2222056. doi:10.1001/jamanetworkopen.2022.22056

eTable 1. Medication Reconciliation: Ontario Drug Benefit Claims for Selected Drug Information Numbers

eTable 2. Canadian Classification of Interventions Procedure Codes for Cardiac Conditions

eTable 3. Congestive Heart Failure and Chronic Obstructive Pulmonary Disease Comorbidities at Index Admission

eTable 4. Additional Congestive Heart Failure Comorbidities at Index Admission

eTable 5. Additional Chronic Obstructive Pulmonary Disease Comorbidities at Index Admission

eTable 6. Postdischarge Physician Visits by Fiscal Years (FYs) 2005-2012 and 2013-2018

eTable 7. Adverse Outcome Rates Overall and by Sex, Length of Stay, and Subgroup at Increased Risk

eTable 8. Cox Proportional Hazards Model for Death Within 30 d Among Patients With Acute Myocardial Infarction

eTable 9. Cox Proportional Hazards Model for All-Cause Readmissions (Death as Competing Risk) Within 30 d Among Individuals With Acute Myocardial Infarction

eTable 10. Cox Proportional Hazards Model for Major Cardiac Event (Death as Competing Risk) Within 30 d Among Individuals With Acute Myocardial Infarction

eTable 11. Cox Proportional Hazards Model for Death Within 90 d Among Individuals With Acute Myocardia Infarction

eTable 12. Cox Proportional Hazards Model for All-Cause Readmissions (Death as Competing Risk) Within 90 d Among Individuals With Acute Myocardial Infarction

eTable 13. Cox Proportional Hazards Model for Major Cardiac Event (Death as Competing Risk) Within 90 d Among Individuals With Acute Myocardial Infarction

eTable 14. Cox Proportional Hazards Model for Death Within 30 d Among Individuals With Congestive Heart Failure

eTable 15. Cox Proportional Hazards Model for All-Cause Readmissions (Death as Competing Risk) Within 30 d Among Individuals With Congestive Heart Failure

eTable 16. Cox Proportional Hazards Model for Major Cardiac Event (Death as Competing Risk) Within 30 d Among Individuals With Congestive Heart Failure

eTable 17. Cox Proportional Hazards Model for Death Within 90 d Among Individuals With Congestive Heart Failure

© 2022 Saxena FE et al. *JAMA Network Open.*

eTable 18. Cox Proportional Hazards Model for All-Cause Readmissions (Death as Competing Risk) Within 90 d Among Individuals With Congestive Heart Failure

eTable 19. Cox Proportional Hazards Model for Major Cardiac Event (Death as Competing Risk) Within 90 d Among Individuals With Congestive Heart Failure

eTable 20. Cox Proportional Hazards Model for Death Within 30 d Among Individuals With Chronic Obstructive Pulmonary Disease

eTable 21. Cox Proportional Hazards Model for All-Cause Readmissions (Death as Competing Risk) Within 30 d Among Individuals With Chronic Obstructive Pulmonary Disease

eTable 22. Cox Proportional Hazards Model for Readmission for Chronic Obstructive Pulmonary Disease (COPD) or COPD-Related Condition (Death as Competing Risk) Within 90 d Among Individuals With COPD

eTable 23. Cox Proportional Hazards Model for Death Within 90 Days Among Individuals With Chronic Obstructive Pulmonary Disease

eTable 24. Cox Proportional Hazards Model for All-Cause Readmission (Death as Competing Risk) Within 90 d Among Individuals With Chronic Obstructive Pulmonary Disease

eTable 25. Cox Proportional Hazards Model for Readmission for Chronic Obstructive Pulmonary Disease (COPD) or COPD-Related Conditions (Death as Competing Risk) Within 90 d Among Individuals With COPD

eTable 26. Adjusted Hazard Ratios With 95% CIs of Adverse Events for Patients Who Received vs Did Not Receive Early Physician Follow-up, Overall, by Sex, Length of Stay Group, and Subgroup at Increased Risk

This supplemental material has been provided by the authors to give readers additional information about their work.

eTable 1. Medication Reconciliation: Ontario Drug Benefit (ODB) Claims for Selected Drug Iinformation Numberss

DIN	Description
93899979	MEDSCHECK
93899981	HOSPITAL DISCHARGE MEDSCHECK FOLLOW UP
93899982	PHARMACISTS DOCUMENTED DECISION MEDSCHECK FOLLOW UP
93899983	PHYSICIAN/RN EC REFERRAL MEDSCHECK FOLLOW UP
93899984	PLANNED HOSPITAL ADMISSION MEDSCHECK FOLLOW UP
93899985	MEDICATION REVIEW
93899986	MEDICATION REVIEW LONG TERM CARE QUARTERLY
93899987	MEDICATION REVIEW HOME
93899988	MEDICATION REVIEW FOR DIABETES
93899989	MEDICATION REVIEW FOR DIABETES- FOLLOW-UP

© 2022 Saxena FE et al. *JAMA Network Open.*

eTable 2. Canadian Classification of Interventions (CCI) Procedure Codes for Cardiac Conditions

**Coronary artery bypass graft surgery (CABG)**	
**CCI**	**Description**
1IJ76	BYPASS, CORONARY ARTERIES
1IJ80	REPAIR, CORONARY ARTERIES
**Percutaneous coronary intervention (PCI)**	
**CCI**	**Description**
1IJ26	BRACHYTHERAPY, CORONARY ARTERIES
1IJ50	DILATION, CORONARY ARTERIES
1IJ55	REMOVAL OF DEVICE, CORONARY ARTERIES
1IJ57	EXTRACTION, CORONARY ARTERIES
**Cardiac catheterization (CATH)**	
**CCI**	**Description**
3IS10	Xray, vena cava (superior and/or inferior)
3IP10	Xray, heart with coronary arteries
2HZ28	Pressure measurement, heart
**Implantable cardiac defibrillator (ICD)**	
**CCI**	**Description**
1HZ53GRFS	Implantation of internal device, heart NEC percutaneous transluminal approach [transvenous] or approach NOS cardioverter/defibrillator [AICD, CRT-D, BiV-ICD]
1HZ53LAFS	Implantation of internal device, heart NEC open approach (thoracotomy) cardioverter/defibrillator [AICD, CRT-D, BiV-ICD]
**Permanent pacemaker (PPM)**	
**CCI**	**Description**
1HZ53GRNM	Implantation of internal device, heart NEC percutaneous transluminal approach [transvenous] or approach NOS Single chamber, programmable, rate responsive pacemaker (VVD, VVI, AAI, VVIR, AAIR modes)
1HZ53LANM	Implantation of internal device, heart NEC open approach (thoracotomy) single chamber programmable rate responsive pacemaker (VVD, VVI, AAI, VVIR, AAIR modes)
1HZ53GRNK	Implantation of internal device, heart NEC percutaneous transluminal approach [transvenous] or approach NOS dual chamber programmable rate responsive pacemaker (DVI, DDD, DDDR modes)
1HZ53LANK	Implantation of internal device, heart NEC open approach [thoracotomy] Dual chamber programmable, rate responsive pacemaker (DVI, DDD, DDDR modes)
1HZ53GRNL	Implantation of internal device, heart NEC percutaneous transluminal approach [transvenous] or approach NOS fixed rate pacemaker (VOO mode)
1HZ53LANL	Implantation of internal device, heart NEC open approach [thoracotomy] Fixed rate pacemaker (VOO mode)
1HZ53GRFR	Implantation of internal device, heart NEC percutaneous transluminal (transvenous) approach or approach NOS cardiac resynchronization therapy pacemaker [CRT-P]
1HZ53LAFR	Implantation of internal device, heart NEC open (thoracotomy) approach cardiac resynchronization pacemaker [CRT-P]

© 2022 Saxena FE et al. *JAMA Network Open.*

eTable 3. Congestive Heart Failure and Chronic Obstructive Pulmonary Disease Comorbidities at Index Admission

ICD-10-CA Codes	Condition
I20-I25	Ischemic heart disease
R57	Shock
I70-I74, I77-I82, R02	Peripheral vascular disease PVD
I44-I49	Arrhythmia
I60-I69	Cerebrovascular disease CVD
I10-I13, I15	Hypertensive disease
F00-F03	Dementia
	Other cardiovascular disease (any of the following):
I30-I33, I40-I43, I51.4	Myo-/peri-/endo-cardial disease
I26-I28	Pulmonary vascular disease
I00-I02, I05-I09	Rheumatic heart disease
R55, R96.0	Syncope, Sudden death
I34-I39	Valvular heart disease
I51, I52, I95, I97	Other cardiac

© 2022 Saxena FE et al. *JAMA Network Open.*

eTable 4. Additional Congestive Heart Failure Comorbidities at Index Admission

**ICD-10-CA Codes**	**Condition**
J20, J40-J45	COPD
N17, N19, R34	Acute renal failure
I12, I13, T824, Z992	Chronic renal failure
E10, E11, E13, E14	Diabetes
C0, C1, C20, C21, C22, C23, C24, C25, C26, C30, C31, C32, C33, C34, C37, C38, C39, C40, C41, C43, C45, C46, C47, C48, C49, C50, C51, C52, C53, C54, C55, C56, C57, C58, C6, C70, C71, C72, C73, C74, C75, C76, C81, C82, C83, C84, C85, C88, C90, C91, C92, C93, C94, C95, C96, C97	Non-metastatic cancer
C77, C78, C79, C80	Metastatic cancer
I850, K704, K721, K729, K766, K767, K778, I859, I982	Moderate/severe liver disease

© 2022 Saxena FE et al. *JAMA Network Open.*

eTable 5. Additional Chronic Obstructive Pulmonary Disease Comorbidities at Index Admission

ICD-10-CA Codes Condition	Condition
I500, I501, or I509	CHF
	Other chronic respiratory condition (any of the following):
E84	Cystic fibrosis
J47	Bronchiectasis
J60	Coalworker's pneumoconiosis
J61	Pneumoconiosis due to asbestos and other mineral fibres
J620	Pneumoconiosis due to talc dust
J628	Pneumoconiosis due to other dust containing silica
J630	Aluminosis (of lung)
J631	Bauxite fibrosis (of lung)
J632	Berylliosis
J633	Graphite fibrosis (of lung)
J634	Siderosis
J635	Stannosis
J638	Pneumoconiosis due to other specified inorganic dusts
J64	Unspecified pneumoconiosis
J65	Pneumoconiosis associated with tuberculosis
J660	Byssinosis
J661	Flax-dresser's disease
J662	Cannabinosis
J668	Airway disease due to other specific organic dusts
J670	Farmer's lung
J671	Bagassosis
J672	Bird fancier's lung
J673	Suberosis
J674	Maltworker's lung
J675	Mushroom-worker's lung
J676	Maple-bark-stripper's lung
J677	Air-conditioner and humidifier lung
J678	Hypersensitivity pneumonitis due to other organic dusts
J6790	Allergic bronchopulmonary aspergillosis
J6799	Hypersensitivity pneumonitis due to unspecified organic dust
J680	Bronchitis and pneumonitis due to chemicals, gases, fumes & vapours
J701	Chronic and other pulmonary manifestations due to radiation
J703	Chronic drug-induced interstitial lung disorders
J704	Drug-induced interstitial lung disorders, unspecified
J708	Respiratory conditions due to other specified external agents
J709	Respiratory conditions due to unspecified external agent
J840	Alveolar and parietoalveolar conditions
J841	Other interstitial pulmonary diseases with fibrosis
J848	Other specified interstitial pulmonary diseases
J849	Interstitial pulmonary disease, unspecified
J850	Gangrene and necrosis of lung
J851	Abscess of lung with pneumonia
J852	Abscess of lung without pneumonia
J860	Pyothorax with fistula
J869	Pyothorax without fistula
J90	Pleural effusion, not elsewhere classified
J91	Pleural effusion in conditions classified elsewhere

© 2022 Saxena FE et al. *JAMA Network Open.*

J920	Pleural plaque with presence of asbestos
**OHIP Codes**	
277	Cystic fibrosis
494	Bronchiectasis
501	Asbestosis
502	Silicosis
511	Pleurisy with or without effusion
515	Pulmonary fibrosis

© 2022 Saxena FE et al. *JAMA Network Open.*

eTable 6. Postdischarge Physician Visits by Fiscal Years (FYs) 2005-2012 and 2013-2018

	AMI		CHF		COPD	
**Physician visits**	**FY 2005 - 2012**	**FY 2013 - 2018**	**FY 2005 - 2012**	**FY 2013 - 2018**	**FY 2005 - 2012**	**FY 2013 - 2018**
	N=107,100	N=91,754	N=68,389	N=64,669	N=67,627	N=51,207
Visit with PCP or relevant specialist within 7 days	45.93%	45.76%	42.42%	42.50%	34.04%	33.46%
Visit with PCP or relevant specialist within 14 days	69.71%	71.48%	64.65%	67.01%	57.36%	58.44%
Visit with PCP or relevant specialist within 30 days	85.82%	86.48%	81.92%	83.60%	76.16%	76.65%
Collaborative care (visit with PCP and relevant specialist) within 30 days	23.00%	24.48%	23.00%	28.65%	11.06%	12.86%
Visit with relevant specialist within 30 days	29.13%	30.10%	31.10%	38.77%	15.75%	18.20%

PCP: primary care provider

© 2022 Saxena FE et al. *JAMA Network Open.*

eTable 7. Adverse Outcome Rates Overall and by Sex, Length of Stay, and Subgroup at Increased Risk

	Overall	Age ≥75 years	Female	Male	LOS 2 days	LOS 3-7 days	LOS > 7 days	Comorbidity	Low SES	Rural
**Cohort Adverse Outcomes**										
**Acute Myocardial Infarction (AMI)**										
Unplanned readmission within 30 days of discharge, %	6.74	10.41	8.49	5.92	3.87	5.98	10.43	7.40	7.12	6.89
Major cardiac event within 30 days of discharge, %	2.44	4.26	3.19	2.08	1.26	2.27	3.58	2.74	2.58	2.65
Death within 30 days of discharge, %	0.71	1.55	0.84	0.64	0.30	0.53	1.38	0.81	0.74	0.71
Unplanned readmission within 90 days of discharge, %	13.67	21.62	17.02	12.09	7.82	12.40	20.44	15.04	14.35	14.06
Major cardiac event within 90 days of discharge, %	5.18	9.26	6.62	4.50	2.66	4.77	7.75	5.83	5.50	5.56
Death within 90 days of discharge, %	2.20	5.04	2.70	1.96	0.85	1.67	4.34	2.55	2.32	2.25
**Congestive Heart Failure (CHF)**										
Unplanned readmission within 30 days of discharge, %	13.52	14.24	13.57	13.48	11.68	12.66	15.40	13.69	13.68	13.55
Major cardiac event within 30 days of discharge, %	5.98	6.41	5.97	5.98	5.52	5.72	6.50	6.05	6.10	5.79
Death within 30 days of discharge, %	2.23	2.77	2.08	2.37	1.34	1.79	3.18	2.25	2.24	2.37
Unplanned readmission within 90 days of discharge, %	29.12	30.98	29.35	28.92	25.88	27.68	32.30	29.53	29.55	29.40
Major cardiac event within 90 days of discharge, %	13.40	14.49	13.43	13.39	12.15	13.03	14.35	13.63	13.70	13.27
Death within 90 days of discharge, %	7.66	9.43	7.24	8.01	5.39	6.46	10.17	7.74	7.60	8.02
**Chronic Obstructive Pulmonary Disease (COPD)**										
Unplanned readmission within 30 days of discharge, %	10.37	11.54	9.45	11.24	8.45	9.78	12.59	10.72	10.45	9.97
Major cardiac event within 30 days of discharge, %	4.42	4.59	3.97	4.84	4.19	4.27	4.84	4.48	4.52	4.10
Death within 30 days of discharge, %	1.71	2.19	1.36	2.06	0.98	1.38	2.80	1.78	1.71	1.68

© 2022 Saxena FE et al. *JAMA Network Open.*

Unplanned readmission within 90 days of discharge, %	22.47	25.13	20.74	24.12	18.88	21.34	26.71	23.20	22.67	21.68
Major cardiac event within 90 days of discharge, %	10.38	10.86	9.50	11.23	9.40	10.16	11.37	10.55	10.58	9.77
Death within 90 days of discharge, %	5.43	7.05	4.37	6.43	3.40	4.58	8.24	5.68	5.41	5.31

© 2022 Saxena FE et al. *JAMA Network Open.*

eTable 8. Cox Proportional Hazards Model for Death Within 30 d Among Patients With Acute Myocardial Infarction

Parameter	Hazard Ratio	95% Hazard Ratio Confidence Limits	
Office visit (PC or relevant specialist) within 7 days after discharge	0.931	0.834	1.038
Females: 46 - 64 (ref= females 20 - 45)	2.622	0.635	10.825
Females: 65 - 74	4.797	1.178	19.542
Females: 75 - 84	4.909	1.211	19.895
Females: 85+	10.242	2.535	41.374
Males: 20 - 45	0.727	0.139	3.806
Males: 46 - 64	2.772	0.684	11.235
Males: 65 - 74	5.26	1.302	21.26
Males 75 - 84	7.369	1.825	29.751
Males: 85+	11.118	2.749	44.964
Income quintile: 1 (ref=income quintile 5)	1.075	0.904	1.277
Income quintile: 2	0.998	0.837	1.19
Income quintile: 3	0.902	0.751	1.084
Income quintile: 4	0.94	0.78	1.132
Income quintile: Missing	0.868	0.331	2.275
Urban (RIO < 10) (ref=rural)	1.041	0.915	1.186
RIO (missing)	0.935	0.564	1.55
Ambulatory visit to a comprehensive PC physician in the past year	1.063	0.885	1.276
Usual provider of care: FHT (ref=no usual provider of care)	0.061	0.048	0.078
Usual provider of care: non-FHT (ref=no usual provider of care)	0.073	0.058	0.091
Specialty of discharging physician: Cardiologist (ref=GP/FP)	0.746	0.636	0.875
Specialty of discharging physician: GIM	0.817	0.701	0.953
Specialty of discharing physician: Other	0.827	0.651	1.052
AMI severity conditions: Shock	2.334	1.479	3.682
AMI severity conditions: CHF	1.912	1.672	2.187
AMI severity conditions: Cancer	4.421	3.614	5.409
AMI severity conditions: CVD	1.845	1.312	2.596
AMI severity conditions: Diabetes with complications	1.108	0.949	1.293
AMI severity conditions: Cardiac dysrhythmias	1.245	1.083	1.432
AMI severity conditions: Acute renal failure	1.363	1.128	1.648
AMI severity conditions: Chronic renal failure	1.414	1.18	1.694
AMI severity conditions: Pulmonary edema	1.324	0.686	2.557
History of AMI in previous 1 - 5 years	1.281	0.925	1.775
History of CHF or angina in previous 1 - 5 years	1.04	0.817	1.324
Cardiac revascularization (CABH/PCI) during index admission	0.318	0.273	0.369
History of PCI or CABG in previous 1 - 5 years	0.763	0.585	0.995
Charlson comorbidity in previous 5 years: Myocardial infarction	0.864	0.636	1.174
Charlson comorbidity in previous 5 years: CHF	1.478	1.139	1.917
Charlson comorbidity in previous 5 years: Peripheral vascular disease	1.238	0.996	1.54
Charlson comorbidity in previous 5 years: Cerebrovascular disease	1.1	0.867	1.394
Charlson comorbidity in previous 5 years: Dementia	1.704	1.274	2.28
Charlson comorbidity in previous 5 years: COPD	1.158	0.967	1.385
Charlson comorbidity in previous 5 years: Connective tissue/rheumatic disease	0.707	0.377	1.326
Charlson comorbidity in previous 5 years: Peptic ulcer disease	1.182	0.857	1.631
Charlson comorbidity in previous 5 years: Mild liver disease	1.774	1.073	2.933
Charlson comorbidity in previous 5 years: Diabetes without complications	1.107	0.889	1.379
Charlson comorbidity in previous 5 years: Diabetes with complications	1.015	0.833	1.236
Charlson comorbidity in previous 5 years: Hemiplegia or paraplegia	1.126	0.622	2.037
Charlson comorbidity in previous 5 years: Renal disease	1.25	1.032	1.514
Charlson comorbidity in previous 5 years: Primary cancer	0.975	0.788	1.207
Charlson comorbidity in previous 5 years: Moderate or severe liver disease	2.068	1.057	4.048
Charlson comorbidity in previous 5 years: Metastatic cancer	1.938	1.437	2.612
Charlson comorbidity in previous 5 years: HIV/AIDS	14.727	4.487	48.338
Year of discharge: 2006 (ref=2005)	0.881	0.668	1.161
Year of discharge: 2007	0.972	0.739	1.279
Year of discharge: 2008	1.19	0.909	1.558
Year of discharge: 2009	1.031	0.775	1.372
Year of discharge: 2010	1.341	1.025	1.755
Year of discharge: 2011	0.943	0.703	1.265
Year of discharge: 2012	1.099	0.816	1.48
Year of discharge: 2013	1.09	0.809	1.469
Year of discharge: 2014	0.399	0.306	0.519
Year of discharge: 2015	1.244	0.93	1.664
Year of discharge: 2016	1.394	1.052	1.847
Year of discharge: 2017	1.444	1.084	1.924
Year of discharge: 2018	1.018	0.746	1.389
Year of discharge: 2019	1.183	0.854	1.64

© 2022 Saxena FE et al. *JAMA Network Open.*

eTable 9. Cox Proportional Hazards Model for All-Cause Readmissions (Death as Competing Risk) Within 30 d Among Individuals With Acute Myocardial Infarction

Parameter	Hazard Ratio	95% Hazard Ratio Confidence Limits	
Office visit (PC or relevant specialist) within 7 days after discharge	0.986	0.952	1.021
Females: 46 - 64 (ref= females 20 - 45)	0.918	0.764	1.104
Females: 65 - 74	1.14	0.949	1.37
Females: 75 - 84	1.25	1.042	1.499
Females: 85+	1.59	1.322	1.913
Males: 20 - 45	0.728	0.594	0.892
Males: 46 - 64	0.756	0.632	0.904
Males: 65 - 74	0.936	0.781	1.121
Males 75 - 84	1.178	0.984	1.412
Males: 85+	1.348	1.117	1.626
Income quintile: 1 (ref=income quintile 5)	1.159	1.095	1.227
Income quintile: 2	1.13	1.067	1.197
Income quintile: 3	1.088	1.026	1.154
Income quintile: 4	1.017	0.957	1.08
Income quintile: Missing	1.133	0.866	1.482
Urban (RIO < 10) (ref=rural)	0.979	0.941	1.019
RIO (missing)	1.204	1.05	1.381
Ambulatory visit to a comprehensive PC physician in the past year	1.179	1.115	1.248
Usual provider of care: FHT (ref=no usual provider of care)	0.459	0.387	0.544
Usual provider of care: non-FHT (ref=no usual provider of care)	0.452	0.382	0.535
Specialty of discharging physician: Cardiologist (ref=GP/FP)	0.901	0.853	0.951
Specialty of discharging physician: GIM	0.877	0.829	0.926
Specialty of discharing physician: Other	1.074	1	1.154
AMI severity conditions: Shock	1.512	1.287	1.777
AMI severity conditions: CHF	1.489	1.417	1.565
AMI severity conditions: Cancer	1.712	1.55	1.891
AMI severity conditions: CVD	1.094	0.933	1.284
AMI severity conditions: Diabetes with complications	1.119	1.065	1.176
AMI severity conditions: Cardiac dysrhythmias	1.185	1.126	1.247
AMI severity conditions: Acute renal failure	1.223	1.131	1.321
AMI severity conditions: Chronic renal failure	1.245	1.153	1.345
AMI severity conditions: Pulmonary edema	1.556	1.241	1.951
History of AMI in previous 1 - 5 years	1.121	1	1.257
History of CHF or angina in previous 1 - 5 years	1.198	1.105	1.298
Cardiac revascularization (CABH/PCI) during index admission	0.747	0.717	0.779
History of PCI or CABG in previous 1 - 5 years	0.941	0.868	1.019
Charlson comorbidity in previous 5 years: Myocardial infarction	0.967	0.869	1.075
Charlson comorbidity in previous 5 years: CHF	1.06	0.97	1.159
Charlson comorbidity in previous 5 years: Peripheral vascular disease	1.16	1.071	1.256
Charlson comorbidity in previous 5 years: Cerebrovascular disease	1.077	0.987	1.174
Charlson comorbidity in previous 5 years: Dementia	1.06	0.909	1.236
Charlson comorbidity in previous 5 years: COPD	1.335	1.251	1.425
Charlson comorbidity in previous 5 years: Connective tissue/rheumatic disease	1.269	1.069	1.507
Charlson comorbidity in previous 5 years: Peptic ulcer disease	1.141	1.008	1.291
Charlson comorbidity in previous 5 years: Mild liver disease	1.245	1	1.552
Charlson comorbidity in previous 5 years: Diabetes without complications	1.155	1.074	1.243
Charlson comorbidity in previous 5 years: Diabetes with complications	1.316	1.233	1.404
Charlson comorbidity in previous 5 years: Hemiplegia or paraplegia	1.249	1.004	1.555
Charlson comorbidity in previous 5 years: Renal disease	1.17	1.08	1.266
Charlson comorbidity in previous 5 years: Primary cancer	1.051	0.971	1.137
Charlson comorbidity in previous 5 years: Moderate or severe liver disease	1.196	0.898	1.592
Charlson comorbidity in previous 5 years: Metastatic cancer	1.283	1.104	1.492
Charlson comorbidity in previous 5 years: HIV/AIDS	1.731	0.799	3.748
Year of discharge: 2006 (ref=2005)	0.861	0.79	0.938
Year of discharge: 2007	0.888	0.815	0.967
Year of discharge: 2008	0.849	0.778	0.927
Year of discharge: 2009	0.805	0.736	0.88
Year of discharge: 2010	0.836	0.765	0.915
Year of discharge: 2011	0.835	0.762	0.915
Year of discharge: 2012	0.849	0.774	0.93
Year of discharge: 2013	0.812	0.741	0.89
Year of discharge: 2014	0.756	0.689	0.83
Year of discharge: 2015	0.817	0.744	0.896
Year of discharge: 2016	0.745	0.678	0.818
Year of discharge: 2017	0.776	0.706	0.853
Year of discharge: 2018	0.753	0.684	0.828
Year of discharge: 2019	0.752	0.677	0.836

© 2022 Saxena FE et al. *JAMA Network Open.*

eTable 10. Cox Proportional Hazards Model for Major Cardiac Event (Death as Competing Risk) Within 30 d Among Individuals With Acute Myocardial Infarction

Parameter	Hazard Ratio	95% Hazard Ratio Confidence Limits	
Office visit (PC or relevant specialist) within 7 days after discharge	1.021	0.963	1.082
Females: 46 - 64 (ref= females 20 - 45)	1.2	0.833	1.73
Females: 65 - 74	1.485	1.032	2.136
Females: 75 - 84	1.912	1.336	2.735
Females: 85+	2.702	1.884	3.876
Males: 20 - 45	0.812	0.541	1.22
Males: 46 - 64	1.058	0.742	1.511
Males: 65 - 74	1.381	0.966	1.974
Males 75 - 84	1.745	1.221	2.494
Males: 85+	2.282	1.585	3.285
Income quintile: 1 (ref=income quintile 5)	1.142	1.041	1.254
Income quintile: 2	1.078	0.981	1.185
Income quintile: 3	1.007	0.913	1.11
Income quintile: 4	0.928	0.839	1.027
Income quintile: Missing	1.193	0.774	1.838
Urban (RIO < 10) (ref=rural)	0.854	0.8	0.911
RIO (missing)	1.093	0.866	1.378
Ambulatory visit to a comprehensive PC physician in the past year	1.173	1.064	1.292
Usual provider of care: FHT (ref=no usual provider of care)	0.467	0.351	0.621
Usual provider of care: non-FHT (ref=no usual provider of care)	0.448	0.338	0.594
Specialty of discharging physician: Cardiologist (ref=GP/FP)	1.075	0.982	1.176
Specialty of discharging physician: GIM	1.031	0.943	1.127
Specialty of discharing physician: Other	0.929	0.815	1.058
AMI severity conditions: Shock	1.505	1.145	1.979
AMI severity conditions: CHF	1.787	1.654	1.931
AMI severity conditions: Cancer	1.231	1.017	1.489
AMI severity conditions: CVD	1.177	0.923	1.501
AMI severity conditions: Diabetes with complications	1.2	1.104	1.304
AMI severity conditions: Cardiac dysrhythmias	1.003	0.921	1.093
AMI severity conditions: Acute renal failure	1.142	1.011	1.291
AMI severity conditions: Chronic renal failure	1.197	1.063	1.349
AMI severity conditions: Pulmonary edema	1.992	1.436	2.763
History of AMI in previous 1 - 5 years	1.079	0.911	1.277
History of CHF or angina in previous 1 - 5 years	1.364	1.206	1.542
Cardiac revascularization (CABH/PCI) during index admission	0.567	0.528	0.609
History of PCI or CABG in previous 1 - 5 years	1.028	0.911	1.16
Charlson comorbidity in previous 5 years: Myocardial infarction	1.09	0.93	1.278
Charlson comorbidity in previous 5 years: CHF	1.064	0.932	1.215
Charlson comorbidity in previous 5 years: Peripheral vascular disease	1.206	1.069	1.361
Charlson comorbidity in previous 5 years: Cerebrovascular disease	1.026	0.898	1.174
Charlson comorbidity in previous 5 years: Dementia	0.999	0.789	1.266
Charlson comorbidity in previous 5 years: COPD	1.039	0.934	1.157
Charlson comorbidity in previous 5 years: Connective tissue/rheumatic disease	1.103	0.824	1.477
Charlson comorbidity in previous 5 years: Peptic ulcer disease	1.038	0.846	1.274
Charlson comorbidity in previous 5 years: Mild liver disease	0.93	0.615	1.408
Charlson comorbidity in previous 5 years: Diabetes without complications	1.227	1.093	1.378
Charlson comorbidity in previous 5 years: Diabetes with complications	1.394	1.257	1.546
Charlson comorbidity in previous 5 years: Hemiplegia or paraplegia	1.145	0.809	1.62
Charlson comorbidity in previous 5 years: Renal disease	1.183	1.048	1.335
Charlson comorbidity in previous 5 years: Primary cancer	0.841	0.733	0.965
Charlson comorbidity in previous 5 years: Moderate or severe liver disease	0.587	0.308	1.118
Charlson comorbidity in previous 5 years: Metastatic cancer	0.824	0.604	1.124
Charlson comorbidity in previous 5 years: HIV/AIDS	2.753	0.871	8.7
Year of discharge: 2006 (ref=2005)	0.771	0.678	0.877
Year of discharge: 2007	0.769	0.674	0.876
Year of discharge: 2008	0.733	0.64	0.839
Year of discharge: 2009	0.621	0.537	0.717
Year of discharge: 2010	0.688	0.597	0.792
Year of discharge: 2011	0.669	0.578	0.775
Year of discharge: 2012	0.711	0.614	0.822
Year of discharge: 2013	0.595	0.51	0.693
Year of discharge: 2014	0.58	0.498	0.677
Year of discharge: 2015	0.624	0.536	0.727
Year of discharge: 2016	0.59	0.507	0.688
Year of discharge: 2017	0.553	0.471	0.65
Year of discharge: 2018	0.487	0.412	0.575
Year of discharge: 2019	0.506	0.419	0.61

© 2022 Saxena FE et al. *JAMA Network Open.*

eTable 11. Cox Proportional Hazards Model for Death Within 90 d Among Individuals With Acute Myocardia Infarction

Parameter	Hazard Ratio	95% Hazard Ratio Confidence Limits	
Office visit (PC or relevant specialist) within 7 days after discharge	0.945	0.888	1.007
Females: 46 - 64 (ref= females 20 - 45)	1.807	0.92	3.549
Females: 65 - 74	3.063	1.571	5.975
Females: 75 - 84	4.188	2.159	8.126
Females: 85+	7.331	3.783	14.207
Males: 20 - 45	0.632	0.286	1.4
Males: 46 - 64	1.552	0.798	3.017
Males: 65 - 74	3.537	1.824	6.86
Males 75 - 84	5.419	2.797	10.499
Males: 85+	8.502	4.383	16.492
Income quintile: 1 (ref=income quintile 5)	1.123	1.015	1.242
Income quintile: 2	1.036	0.935	1.149
Income quintile: 3	0.993	0.894	1.104
Income quintile: 4	0.993	0.892	1.105
Income quintile: Missing	1.084	0.637	1.843
Urban (RIO < 10) (ref=rural)	1.027	0.955	1.105
RIO (missing)	1.068	0.822	1.388
Ambulatory visit to a comprehensive PC physician in the past year	1.106	0.99	1.235
Usual provider of care: FHT (ref=no usual provider of care)	0.061	0.052	0.073
Usual provider of care: non-FHT (ref=no usual provider of care)	0.066	0.056	0.078
Specialty of discharging physician: Cardiologist (ref=GP/FP)	0.754	0.688	0.826
Specialty of discharging physician: GIM	0.826	0.757	0.902
Specialty of discharing physician: Other	0.815	0.709	0.937
AMI severity conditions: Shock	2.073	1.564	2.748
AMI severity conditions: CHF	1.872	1.735	2.02
AMI severity conditions: Cancer	4.02	3.558	4.542
AMI severity conditions: CVD	1.444	1.158	1.803
AMI severity conditions: Diabetes with complications	1.243	1.136	1.361
AMI severity conditions: Cardiac dysrhythmias	1.199	1.106	1.301
AMI severity conditions: Acute renal failure	1.421	1.273	1.587
AMI severity conditions: Chronic renal failure	1.378	1.235	1.539
AMI severity conditions: Pulmonary edema	1.64	1.135	2.368
History of AMI in previous 1 - 5 years	1.173	0.987	1.395
History of CHF or angina in previous 1 - 5 years	1.064	0.929	1.219
Cardiac revascularization (CABH/PCI) during index admission	0.353	0.324	0.385
History of PCI or CABG in previous 1 - 5 years	0.744	0.639	0.866
Charlson comorbidity in previous 5 years: Myocardial infarction	0.982	0.835	1.155
Charlson comorbidity in previous 5 years: CHF	1.36	1.178	1.571
Charlson comorbidity in previous 5 years: Peripheral vascular disease	1.367	1.209	1.544
Charlson comorbidity in previous 5 years: Cerebrovascular disease	1.162	1.017	1.326
Charlson comorbidity in previous 5 years: Dementia	1.487	1.241	1.781
Charlson comorbidity in previous 5 years: COPD	1.368	1.239	1.511
Charlson comorbidity in previous 5 years: Connective tissue/rheumatic disease	1.254	0.944	1.668
Charlson comorbidity in previous 5 years: Peptic ulcer disease	1.047	0.862	1.272
Charlson comorbidity in previous 5 years: Mild liver disease	1.502	1.066	2.116
Charlson comorbidity in previous 5 years: Diabetes without complications	1.053	0.928	1.194
Charlson comorbidity in previous 5 years: Diabetes with complications	1.108	0.991	1.239
Charlson comorbidity in previous 5 years: Hemiplegia or paraplegia	1.011	0.706	1.45
Charlson comorbidity in previous 5 years: Renal disease	1.238	1.104	1.389
Charlson comorbidity in previous 5 years: Primary cancer	1.029	0.911	1.163
Charlson comorbidity in previous 5 years: Moderate or severe liver disease	2.06	1.383	3.069
Charlson comorbidity in previous 5 years: Metastatic cancer	2.271	1.899	2.716
Charlson comorbidity in previous 5 years: HIV/AIDS	8.74	3.348	22.812
Year of discharge: 2006 (ref=2005)	0.969	0.832	1.128
Year of discharge: 2007	0.957	0.82	1.116
Year of discharge: 2008	1.043	0.893	1.218
Year of discharge: 2009	1.053	0.899	1.233
Year of discharge: 2010	1.064	0.907	1.249
Year of discharge: 2011	0.997	0.846	1.174
Year of discharge: 2012	1.006	0.85	1.191
Year of discharge: 2013	0.995	0.842	1.176
Year of discharge: 2014	0.43	0.364	0.508
Year of discharge: 2015	1.145	0.971	1.349
Year of discharge: 2016	1.136	0.964	1.34
Year of discharge: 2017	1.165	0.986	1.377
Year of discharge: 2018	0.908	0.761	1.085
Year of discharge: 2019	0.925	0.757	1.129

© 2022 Saxena FE et al. *JAMA Network Open.*

eTable 12. Cox Proportional Hazards Model for All-Cause Readmissions (Death as Competing Risk) Within 90 d Among Individuals With Acute Myocardial Infarction

Parameter	Hazard Ratio	95% Hazard Ratio Confidence Limits	
Office visit (PC or relevant specialist) within 7 days after discharge	0.994	0.969	1.018
Females: 46 - 64 (ref= females 20 - 45)	0.939	0.823	1.071
Females: 65 - 74	1.122	0.984	1.28
Females: 75 - 84	1.34	1.177	1.525
Females: 85+	1.687	1.478	1.925
Males: 20 - 45	0.744	0.643	0.861
Males: 46 - 64	0.758	0.667	0.862
Males: 65 - 74	1	0.879	1.138
Males 75 - 84	1.268	1.115	1.443
Males: 85+	1.54	1.347	1.76
Income quintile: 1 (ref=income quintile 5)	1.153	1.108	1.2
Income quintile: 2	1.107	1.064	1.153
Income quintile: 3	1.068	1.025	1.113
Income quintile: 4	1.029	0.986	1.073
Income quintile: Missing	1.088	0.899	1.317
Urban (RIO < 10) (ref=rural)	0.975	0.948	1.003
RIO (missing)	1.234	1.121	1.358
Ambulatory visit to a comprehensive PC physician in the past year	1.141	1.097	1.187
Usual provider of care: FHT (ref=no usual provider of care)	0.47	0.413	0.534
Usual provider of care: non-FHT (ref=no usual provider of care)	0.466	0.41	0.529
Specialty of discharging physician: Cardiologist (ref=GP/FP)	0.878	0.846	0.912
Specialty of discharging physician: GIM	0.891	0.858	0.926
Specialty of discharing physician: Other	1	0.95	1.052
AMI severity conditions: Shock	1.433	1.269	1.619
AMI severity conditions: CHF	1.472	1.422	1.525
AMI severity conditions: Cancer	1.732	1.613	1.86
AMI severity conditions: CVD	1.082	0.967	1.212
AMI severity conditions: Diabetes with complications	1.139	1.1	1.18
AMI severity conditions: Cardiac dysrhythmias	1.162	1.121	1.205
AMI severity conditions: Acute renal failure	1.231	1.165	1.299
AMI severity conditions: Chronic renal failure	1.193	1.129	1.26
AMI severity conditions: Pulmonary edema	1.429	1.205	1.693
History of AMI in previous 1 - 5 years	1.053	0.974	1.139
History of CHF or angina in previous 1 - 5 years	1.217	1.151	1.286
Cardiac revascularization (CABH/PCI) during index admission	0.713	0.693	0.734
History of PCI or CABG in previous 1 - 5 years	0.943	0.893	0.997
Charlson comorbidity in previous 5 years: Myocardial infarction	1.033	0.961	1.111
Charlson comorbidity in previous 5 years: CHF	1.112	1.046	1.182
Charlson comorbidity in previous 5 years: Peripheral vascular disease	1.193	1.129	1.261
Charlson comorbidity in previous 5 years: Cerebrovascular disease	1.101	1.037	1.169
Charlson comorbidity in previous 5 years: Dementia	1.138	1.028	1.259
Charlson comorbidity in previous 5 years: COPD	1.359	1.298	1.422
Charlson comorbidity in previous 5 years: Connective tissue/rheumatic disease	1.344	1.195	1.511
Charlson comorbidity in previous 5 years: Peptic ulcer disease	1.227	1.128	1.334
Charlson comorbidity in previous 5 years: Mild liver disease	1.363	1.172	1.584
Charlson comorbidity in previous 5 years: Diabetes without complications	1.181	1.122	1.243
Charlson comorbidity in previous 5 years: Diabetes with complications	1.38	1.319	1.445
Charlson comorbidity in previous 5 years: Hemiplegia or paraplegia	1.189	1.022	1.384
Charlson comorbidity in previous 5 years: Renal disease	1.304	1.235	1.377
Charlson comorbidity in previous 5 years: Primary cancer	1.056	0.999	1.115
Charlson comorbidity in previous 5 years: Moderate or severe liver disease	1.354	1.12	1.637
Charlson comorbidity in previous 5 years: Metastatic cancer	1.358	1.221	1.51
Charlson comorbidity in previous 5 years: HIV/AIDS	2.078	1.248	3.462
Year of discharge: 2006 (ref=2005)	0.932	0.877	0.99
Year of discharge: 2007	0.908	0.854	0.966
Year of discharge: 2008	0.88	0.827	0.937
Year of discharge: 2009	0.857	0.804	0.914
Year of discharge: 2010	0.892	0.837	0.95
Year of discharge: 2011	0.889	0.833	0.949
Year of discharge: 2012	0.888	0.832	0.948
Year of discharge: 2013	0.894	0.839	0.954
Year of discharge: 2014	0.795	0.744	0.849
Year of discharge: 2015	0.872	0.816	0.93
Year of discharge: 2016	0.808	0.756	0.863
Year of discharge: 2017	0.808	0.755	0.864
Year of discharge: 2018	0.761	0.711	0.816
Year of discharge: 2019	0.797	0.74	0.859

© 2022 Saxena FE et al. *JAMA Network Open.*

eTable 13. Cox Proportional Hazards Model for Major Cardiac Event (Death as Competing Risk) Within 90 d Among Individuals With Acute Myocardial Infarction

Parameter	Hazard Ratio	95% Hazard Ratio Confidence Limits	
Office visit (PC or relevant specialist) within 7 days after discharge	1.018	0.978	1.059
Females: 46 - 64 (ref= females 20 - 45)	1.151	0.891	1.487
Females: 65 - 74	1.478	1.146	1.907
Females: 75 - 84	1.896	1.474	2.438
Females: 85+	2.641	2.05	3.401
Males: 20 - 45	0.825	0.621	1.097
Males: 46 - 64	1.01	0.786	1.296
Males: 65 - 74	1.405	1.094	1.806
Males 75 - 84	1.86	1.448	2.389
Males: 85+	2.52	1.953	3.251
Income quintile: 1 (ref=income quintile 5)	1.185	1.111	1.265
Income quintile: 2	1.145	1.071	1.223
Income quintile: 3	1.06	0.99	1.135
Income quintile: 4	1.018	0.95	1.091
Income quintile: Missing	1.351	1.002	1.822
Urban (RIO < 10) (ref=rural)	0.88	0.841	0.921
RIO (missing)	1.077	0.918	1.264
Ambulatory visit to a comprehensive PC physician in the past year	1.109	1.038	1.184
Usual provider of care: FHT (ref=no usual provider of care)	0.447	0.364	0.549
Usual provider of care: non-FHT (ref=no usual provider of care)	0.433	0.353	0.53
Specialty of discharging physician: Cardiologist (ref=GP/FP)	1.01	0.95	1.074
Specialty of discharging physician: GIM	0.986	0.928	1.048
Specialty of discharing physician: Other	0.86	0.785	0.941
AMI severity conditions: Shock	1.581	1.316	1.899
AMI severity conditions: CHF	1.878	1.782	1.979
AMI severity conditions: Cancer	1.313	1.155	1.493
AMI severity conditions: CVD	1.128	0.953	1.336
AMI severity conditions: Diabetes with complications	1.255	1.185	1.33
AMI severity conditions: Cardiac dysrhythmias	1.063	1.004	1.126
AMI severity conditions: Acute renal failure	1.188	1.094	1.29
AMI severity conditions: Chronic renal failure	1.137	1.046	1.235
AMI severity conditions: Pulmonary edema	1.87	1.469	2.382
History of AMI in previous 1 - 5 years	1.112	0.994	1.244
History of CHF or angina in previous 1 - 5 years	1.494	1.374	1.625
Cardiac revascularization (CABH/PCI) during index admission	0.584	0.556	0.614
History of PCI or CABG in previous 1 - 5 years	1.011	0.931	1.097
Charlson comorbidity in previous 5 years: Myocardial infarction	1.095	0.985	1.218
Charlson comorbidity in previous 5 years: CHF	1.103	1.008	1.206
Charlson comorbidity in previous 5 years: Peripheral vascular disease	1.199	1.104	1.303
Charlson comorbidity in previous 5 years: Cerebrovascular disease	1.073	0.979	1.175
Charlson comorbidity in previous 5 years: Dementia	1.067	0.913	1.248
Charlson comorbidity in previous 5 years: COPD	1.087	1.012	1.168
Charlson comorbidity in previous 5 years: Connective tissue/rheumatic disease	1.01	0.821	1.242
Charlson comorbidity in previous 5 years: Peptic ulcer disease	1.038	0.904	1.192
Charlson comorbidity in previous 5 years: Mild liver disease	1.035	0.792	1.351
Charlson comorbidity in previous 5 years: Diabetes without complications	1.156	1.065	1.254
Charlson comorbidity in previous 5 years: Diabetes with complications	1.397	1.3	1.501
Charlson comorbidity in previous 5 years: Hemiplegia or paraplegia	1.103	0.871	1.397
Charlson comorbidity in previous 5 years: Renal disease	1.189	1.095	1.291
Charlson comorbidity in previous 5 years: Primary cancer	0.851	0.775	0.934
Charlson comorbidity in previous 5 years: Moderate or severe liver disease	0.932	0.66	1.316
Charlson comorbidity in previous 5 years: Metastatic cancer	0.87	0.707	1.07
Charlson comorbidity in previous 5 years: HIV/AIDS	2.645	1.099	6.363
Year of discharge: 2006 (ref=2005)	0.845	0.772	0.926
Year of discharge: 2007	0.845	0.77	0.927
Year of discharge: 2008	0.792	0.719	0.872
Year of discharge: 2009	0.758	0.687	0.836
Year of discharge: 2010	0.763	0.69	0.843
Year of discharge: 2011	0.756	0.683	0.838
Year of discharge: 2012	0.754	0.68	0.835
Year of discharge: 2013	0.717	0.647	0.795
Year of discharge: 2014	0.628	0.564	0.7
Year of discharge: 2015	0.701	0.631	0.78
Year of discharge: 2016	0.639	0.574	0.712
Year of discharge: 2017	0.623	0.558	0.696
Year of discharge: 2018	0.541	0.482	0.607
Year of discharge: 2019	0.538	0.472	0.613

© 2022 Saxena FE et al. *JAMA Network Open.*

eTable 14. Cox Proportional Hazards Model for Death Within 30 d Among Individuals With Congestive Heart Failure

Parameter	Hazard Ratio	95% Hazard Ratio Confidence Limits	
Office visit (PC or relevant specialist) within 7 days after discharge	0.976	0.905	1.052
Females: 46 - 64 (ref= females 20 - 45)	0.856	0.464	1.578
Females: 65 - 74	1.067	0.589	1.932
Females: 75 - 84	1.461	0.816	2.618
Females: 85+	2.345	1.311	4.192
Males: 20 - 45	0.298	0.118	0.752
Males: 46 - 64	0.729	0.4	1.329
Males: 65 - 74	1.382	0.77	2.482
Males 75 - 84	1.832	1.025	3.274
Males: 85+	2.799	1.564	5.008
Income quintile: 1 (ref=income quintile 5)	1.048	0.928	1.184
Income quintile: 2	1.035	0.916	1.17
Income quintile: 3	1.017	0.897	1.153
Income quintile: 4	0.956	0.839	1.089
Income quintile: Missing	0.681	0.314	1.475
Urban (RIO < 10) (ref=rural)	0.938	0.854	1.029
RIO (missing)	0.872	0.612	1.242
Ambulatory visit to a comprehensive PC physician in the past year	0.986	0.851	1.143
Usual provider of care: FHT (ref=no usual provider of care)	0.084	0.071	0.098
Usual provider of care: non-FHT (ref=no usual provider of care)	0.08	0.069	0.093
Specialty of discharging physician: Cardiologist (ref=GP/FP)	0.795	0.704	0.897
Specialty of discharging physician: GIM	0.945	0.857	1.042
Specialty of discharing physician: Other	0.943	0.811	1.098
CHF severity conditions: Ischemic heart disease	1.036	0.945	1.135
CHF severity conditions: Other cardiovascular disease	1.401	1.281	1.533
CHF severity conditions: Shock	1.172	0.619	2.22
CHF severity conditions: Peripheral vascular disease	1.407	1.148	1.725
CHF severity conditions: Arrhythmia	0.946	0.871	1.027
CHF severity conditions: Cerebrovascular disease	1.085	0.765	1.538
CHF severity conditions: Hypertension	0.767	0.702	0.838
CHF severity conditions: COPD	1.116	1.001	1.244
CHF severity conditions: Dementia	1.596	1.297	1.963
CHF severity conditions: Acute renal failure	1.537	1.388	1.702
CHF severity conditions: Chronic renal failure	1.22	0.979	1.519
CHF severity conditions: Diabetes	1.059	0.946	1.185
CHF severity conditions: Non-metastatic cancer	2.105	1.784	2.484
CHF severity conditions: Metastatic cancer	2.446	1.855	3.226
CHF severity conditions: Moderate/severe liver disease	2.247	1.359	3.716
History of AMI in previous 5 years	1.184	0.981	1.43
History of CHF or angina in previous 5 years	0.972	0.833	1.134
History of implantable cardiac defibrillator in previous 3 years	1.057	0.903	1.236
History of permanent pacemake in previous 3 years	1.062	0.334	3.381
Charlson comorbidity in previous 5 years: Myocardial infarction	1.034	0.862	1.241
Charlson comorbidity in previous 5 years: CHF	1.433	1.232	1.667
Charlson comorbidity in previous 5 years: Peripheral vascular disease	1.235	1.081	1.413
Charlson comorbidity in previous 5 years: Cerebrovascular disease	0.999	0.862	1.159
Charlson comorbidity in previous 5 years: Dementia	1.372	1.124	1.676
Charlson comorbidity in previous 5 years: COPD	1.196	1.077	1.329
Charlson comorbidity in previous 5 years: Connective tissue/rheumatic disease	1.617	1.244	2.102
Charlson comorbidity in previous 5 years: Peptic ulcer disease	0.84	0.676	1.043
Charlson comorbidity in previous 5 years: Mild liver disease	1.502	1.11	2.032
Charlson comorbidity in previous 5 years: Diabetes without complications	0.891	0.754	1.053
Charlson comorbidity in previous 5 years: Diabetes with complications	0.932	0.823	1.056
Charlson comorbidity in previous 5 years: Hemiplegia or paraplegia	1.361	0.974	1.902
Charlson comorbidity in previous 5 years: Renal disease	1.084	0.968	1.213
Charlson comorbidity in previous 5 years: Primary cancer	1.211	1.062	1.382
Charlson comorbidity in previous 5 years: Moderate or severe liver disease	1.71	1.198	2.442
Charlson comorbidity in previous 5 years: Metastatic cancer	1.835	1.433	2.349
Charlson comorbidity in previous 5 years: HIV/AIDS	0	0	0
Year of discharge: 2006 (ref=2005)	1.091	0.9	1.324
Year of discharge: 2007	1.039	0.846	1.275
Year of discharge: 2008	1.158	0.949	1.413
Year of discharge: 2009	1.253	1.026	1.531
Year of discharge: 2010	1.016	0.825	1.251
Year of discharge: 2011	1.021	0.826	1.262
Year of discharge: 2012	1.001	0.812	1.234
Year of discharge: 2013	1.01	0.823	1.239
Year of discharge: 2014	0.346	0.286	0.417
Year of discharge: 2015	0.955	0.78	1.17
Year of discharge: 2016	0.971	0.795	1.186
Year of discharge: 2017	1.067	0.878	1.296
Year of discharge: 2018	1.021	0.838	1.243
Year of discharge: 2019	0.899	0.725	1.115

© 2022 Saxena FE et al. *JAMA Network Open.*

eTable 15. Cox Proportional Hazards Model for All-Cause Readmissions (Death as Competing Risk) Within 30 d Among Individuals With Congestive Heart Failure

Parameter	Hazard Ratio	95% Hazard Ratio Confidence Limits	
Office visit (PC or relevant specialist) within 7 days after discharge	1.006	0.976	1.037
Females: 46 - 64 (ref= females 20 - 45)	0.934	0.764	1.141
Females: 65 - 74	0.972	0.799	1.183
Females: 75 - 84	1.05	0.865	1.273
Females: 85+	1.131	0.932	1.373
Males: 20 - 45	0.762	0.592	0.981
Males: 46 - 64	0.871	0.716	1.059
Males: 65 - 74	0.969	0.797	1.176
Males 75 - 84	1.054	0.869	1.278
Males: 85+	1.119	0.921	1.36
Income quintile: 1 (ref=income quintile 5)	1.075	1.023	1.129
Income quintile: 2	1.022	0.972	1.075
Income quintile: 3	1.012	0.961	1.065
Income quintile: 4	1.011	0.959	1.067
Income quintile: Missing	1.066	0.831	1.368
Urban (RIO < 10) (ref=rural)	1.027	0.989	1.066
RIO (missing)	1.025	0.894	1.174
Ambulatory visit to a comprehensive PC physician in the past year	1.074	1.012	1.141
Usual provider of care: FHT (ref=no usual provider of care)	0.433	0.386	0.487
Usual provider of care: non-FHT (ref=no usual provider of care)	0.426	0.38	0.478
Specialty of discharging physician: Cardiologist (ref=GP/FP)	0.852	0.812	0.894
Specialty of discharging physician: GIM	0.974	0.936	1.013
Specialty of discharing physician: Other	0.975	0.917	1.037
CHF severity conditions: Ischemic heart disease	1.014	0.976	1.053
CHF severity conditions: Other cardiovascular disease	1.12	1.078	1.164
CHF severity conditions: Shock	0.87	0.648	1.168
CHF severity conditions: Peripheral vascular disease	1.051	0.956	1.155
CHF severity conditions: Arrhythmia	1.005	0.972	1.039
CHF severity conditions: Cerebrovascular disease	1.024	0.886	1.184
CHF severity conditions: Hypertension	0.898	0.868	0.93
CHF severity conditions: COPD	1.064	1.017	1.112
CHF severity conditions: Dementia	1.043	0.939	1.159
CHF severity conditions: Acute renal failure	1.314	1.258	1.374
CHF severity conditions: Chronic renal failure	1.167	1.071	1.271
CHF severity conditions: Diabetes	1.03	0.983	1.079
CHF severity conditions: Non-metastatic cancer	1.289	1.187	1.401
CHF severity conditions: Metastatic cancer	1.434	1.225	1.679
CHF severity conditions: Moderate/severe liver disease	1.416	1.104	1.816
History of AMI in previous 5 years	1.091	1.009	1.18
History of CHF or angina in previous 5 years	1.155	1.089	1.225
History of implantable cardiac defibrillator in previous 3 years	1.044	0.979	1.112
History of permanent pacemake in previous 3 years	1.35	0.903	2.019
Charlson comorbidity in previous 5 years: Myocardial infarction	1.005	0.932	1.083
Charlson comorbidity in previous 5 years: CHF	1.179	1.112	1.249
Charlson comorbidity in previous 5 years: Peripheral vascular disease	1.082	1.023	1.145
Charlson comorbidity in previous 5 years: Cerebrovascular disease	1.089	1.027	1.154
Charlson comorbidity in previous 5 years: Dementia	1.077	0.981	1.183
Charlson comorbidity in previous 5 years: COPD	1.238	1.187	1.291
Charlson comorbidity in previous 5 years: Connective tissue/rheumatic disease	1.247	1.112	1.399
Charlson comorbidity in previous 5 years: Peptic ulcer disease	0.998	0.92	1.084
Charlson comorbidity in previous 5 years: Mild liver disease	1.276	1.131	1.439
Charlson comorbidity in previous 5 years: Diabetes without complications	1.019	0.955	1.088
Charlson comorbidity in previous 5 years: Diabetes with complications	1.126	1.069	1.185
Charlson comorbidity in previous 5 years: Hemiplegia or paraplegia	1.058	0.915	1.223
Charlson comorbidity in previous 5 years: Renal disease	1.255	1.2	1.313
Charlson comorbidity in previous 5 years: Primary cancer	1.097	1.035	1.163
Charlson comorbidity in previous 5 years: Moderate or severe liver disease	1.602	1.387	1.851
Charlson comorbidity in previous 5 years: Metastatic cancer	1.136	1.002	1.287
Charlson comorbidity in previous 5 years: HIV/AIDS	2.15	1.254	3.687
Year of discharge: 2006 (ref=2005)	0.968	0.896	1.045
Year of discharge: 2007	0.923	0.852	1.001
Year of discharge: 2008	0.926	0.855	1.004
Year of discharge: 2009	1.017	0.938	1.102
Year of discharge: 2010	0.994	0.917	1.077
Year of discharge: 2011	1.002	0.924	1.088
Year of discharge: 2012	0.997	0.919	1.082
Year of discharge: 2013	1.013	0.936	1.097
Year of discharge: 2014	0.833	0.766	0.905
Year of discharge: 2015	0.948	0.876	1.026
Year of discharge: 2016	0.948	0.876	1.025
Year of discharge: 2017	0.933	0.863	1.009
Year of discharge: 2018	0.916	0.847	0.991
Year of discharge: 2019	0.924	0.848	1.006

© 2022 Saxena FE et al. *JAMA Network Open.*

eTable 16. Cox Proportional Hazards Model for Major Cardiac Event (Death as Competing Risk) Within 30 d Among Individuals With Congestive Heart Failure

Parameter	Hazard Ratio	95% Hazard Ratio Confidence Limits	
Office visit (PC or relevant specialist) within 7 days after discharge	1.021	0.975	1.068
Females: 46 - 64 (ref= females 20 - 45)	1.064	0.762	1.486
Females: 65 - 74	1.143	0.824	1.585
Females: 75 - 84	1.355	0.982	1.87
Females: 85+	1.568	1.136	2.164
Males: 20 - 45	1.085	0.73	1.614
Males: 46 - 64	1.027	0.741	1.423
Males: 65 - 74	1.286	0.931	1.777
Males 75 - 84	1.326	0.961	1.829
Males: 85+	1.505	1.088	2.082
Income quintile: 1 (ref=income quintile 5)	1.125	1.044	1.213
Income quintile: 2	1.055	0.977	1.139
Income quintile: 3	1.056	0.977	1.142
Income quintile: 4	1.03	0.95	1.117
Income quintile: Missing	1.051	0.72	1.534
Urban (RIO < 10) (ref=rural)	1.049	0.991	1.111
RIO (missing)	1.08	0.881	1.323
Ambulatory visit to a comprehensive PC physician in the past year	1.067	0.975	1.168
Usual provider of care: FHT (ref=no usual provider of care)	0.505	0.419	0.609
Usual provider of care: non-FHT (ref=no usual provider of care)	0.504	0.419	0.605
Specialty of discharging physician: Cardiologist (ref=GP/FP)	0.881	0.82	0.946
Specialty of discharging physician: GIM	1	0.942	1.061
Specialty of discharing physician: Other	0.866	0.785	0.956
CHF severity conditions: Ischemic heart disease	1.106	1.046	1.169
CHF severity conditions: Other cardiovascular disease	1.204	1.138	1.275
CHF severity conditions: Shock	1.031	0.686	1.548
CHF severity conditions: Peripheral vascular disease	0.984	0.851	1.137
CHF severity conditions: Arrhythmia	0.999	0.951	1.05
CHF severity conditions: Cerebrovascular disease	0.813	0.642	1.029
CHF severity conditions: Hypertension	0.92	0.874	0.969
CHF severity conditions: COPD	0.984	0.917	1.055
CHF severity conditions: Dementia	0.933	0.793	1.098
CHF severity conditions: Acute renal failure	1.378	1.291	1.47
CHF severity conditions: Chronic renal failure	1.164	1.026	1.32
CHF severity conditions: Diabetes	1.087	1.013	1.167
CHF severity conditions: Non-metastatic cancer	1.142	0.998	1.308
CHF severity conditions: Metastatic cancer	1.065	0.803	1.414
CHF severity conditions: Moderate/severe liver disease	0.633	0.355	1.128
History of AMI in previous 5 years	1.236	1.102	1.386
History of CHF or angina in previous 5 years	1.153	1.056	1.259
History of implantable cardiac defibrillator in previous 3 years	1.055	0.961	1.157
History of permanent pacemake in previous 3 years	1.399	0.802	2.442
Charlson comorbidity in previous 5 years: Myocardial infarction	0.996	0.891	1.113
Charlson comorbidity in previous 5 years: CHF	1.35	1.239	1.472
Charlson comorbidity in previous 5 years: Peripheral vascular disease	1.114	1.024	1.212
Charlson comorbidity in previous 5 years: Cerebrovascular disease	1.159	1.065	1.262
Charlson comorbidity in previous 5 years: Dementia	0.956	0.826	1.105
Charlson comorbidity in previous 5 years: COPD	1.094	1.025	1.168
Charlson comorbidity in previous 5 years: Connective tissue/rheumatic disease	1.009	0.835	1.221
Charlson comorbidity in previous 5 years: Peptic ulcer disease	0.98	0.865	1.109
Charlson comorbidity in previous 5 years: Mild liver disease	1.185	0.98	1.432
Charlson comorbidity in previous 5 years: Diabetes without complications	1.042	0.945	1.149
Charlson comorbidity in previous 5 years: Diabetes with complications	1.116	1.033	1.206
Charlson comorbidity in previous 5 years: Hemiplegia or paraplegia	1.044	0.843	1.292
Charlson comorbidity in previous 5 years: Renal disease	1.133	1.058	1.213
Charlson comorbidity in previous 5 years: Primary cancer	0.991	0.904	1.086
Charlson comorbidity in previous 5 years: Moderate or severe liver disease	1.021	0.781	1.336
Charlson comorbidity in previous 5 years: Metastatic cancer	0.964	0.781	1.19
Charlson comorbidity in previous 5 years: HIV/AIDS	2.944	1.464	5.918
Year of discharge: 2006 (ref=2005)	0.912	0.815	1.021
Year of discharge: 2007	0.9	0.801	1.011
Year of discharge: 2008	0.886	0.787	0.997
Year of discharge: 2009	0.958	0.851	1.078
Year of discharge: 2010	0.981	0.872	1.103
Year of discharge: 2011	0.883	0.781	0.998
Year of discharge: 2012	0.904	0.802	1.02
Year of discharge: 2013	0.938	0.835	1.054
Year of discharge: 2014	0.735	0.647	0.834
Year of discharge: 2015	0.827	0.735	0.931
Year of discharge: 2016	0.859	0.764	0.965
Year of discharge: 2017	0.816	0.726	0.917
Year of discharge: 2018	0.789	0.7	0.888
Year of discharge: 2019	0.813	0.715	0.924

© 2022 Saxena FE et al. *JAMA Network Open.*

eTable 17. Cox Proportional Hazards Model for Death Within 90 d Among Individuals With Congestive Heart Failure

Parameter	Hazard Ratio	95% Hazard Ratio Confidence Limits	
Office visit (PC or relevant specialist) within 7 days after discharge	0.934	0.897	0.973
Females: 46 - 64 (ref= females 20 - 45)	0.896	0.646	1.243
Females: 65 - 74	1.109	0.808	1.524
Females: 75 - 84	1.545	1.131	2.11
Females: 85+	2.422	1.775	3.306
Males: 20 - 45	0.58	0.376	0.896
Males: 46 - 64	0.833	0.605	1.147
Males: 65 - 74	1.339	0.979	1.833
Males 75 - 84	1.865	1.367	2.545
Males: 85+	2.81	2.058	3.837
Income quintile: 1 (ref=income quintile 5)	1.074	1.006	1.147
Income quintile: 2	1.032	0.965	1.102
Income quintile: 3	0.989	0.924	1.059
Income quintile: 4	1.031	0.962	1.106
Income quintile: Missing	0.991	0.693	1.417
Urban (RIO < 10) (ref=rural)	0.947	0.901	0.996
RIO (missing)	0.873	0.718	1.061
Ambulatory visit to a comprehensive PC physician in the past year	1.035	0.955	1.121
Usual provider of care: FHT (ref=no usual provider of care)	0.09	0.081	0.101
Usual provider of care: non-FHT (ref=no usual provider of care)	0.085	0.077	0.094
Specialty of discharging physician: Cardiologist (ref=GP/FP)	0.823	0.771	0.879
Specialty of discharging physician: GIM	0.993	0.942	1.046
Specialty of discharing physician: Other	0.929	0.855	1.01
CHF severity conditions: Ischemic heart disease	1.009	0.959	1.061
CHF severity conditions: Other cardiovascular disease	1.332	1.267	1.4
CHF severity conditions: Shock	1.414	1	1.999
CHF severity conditions: Peripheral vascular disease	1.271	1.13	1.429
CHF severity conditions: Arrhythmia	0.922	0.882	0.964
CHF severity conditions: Cerebrovascular disease	0.987	0.81	1.203
CHF severity conditions: Hypertension	0.777	0.741	0.814
CHF severity conditions: COPD	1.138	1.073	1.208
CHF severity conditions: Dementia	1.482	1.314	1.671
CHF severity conditions: Acute renal failure	1.485	1.403	1.572
CHF severity conditions: Chronic renal failure	1.238	1.101	1.392
CHF severity conditions: Diabetes	0.998	0.938	1.063
CHF severity conditions: Non-metastatic cancer	1.916	1.745	2.105
CHF severity conditions: Metastatic cancer	2.684	2.291	3.145
CHF severity conditions: Moderate/severe liver disease	2.085	1.534	2.833
History of AMI in previous 5 years	1.134	1.021	1.26
History of CHF or angina in previous 5 years	1.044	0.962	1.135
History of implantable cardiac defibrillator in previous 3 years	0.982	0.9	1.071
History of permanent pacemake in previous 3 years	1.629	1.034	2.567
Charlson comorbidity in previous 5 years: Myocardial infarction	0.96	0.868	1.061
Charlson comorbidity in previous 5 years: CHF	1.357	1.251	1.471
Charlson comorbidity in previous 5 years: Peripheral vascular disease	1.238	1.151	1.331
Charlson comorbidity in previous 5 years: Cerebrovascular disease	1.04	0.962	1.125
Charlson comorbidity in previous 5 years: Dementia	1.23	1.096	1.381
Charlson comorbidity in previous 5 years: COPD	1.141	1.077	1.208
Charlson comorbidity in previous 5 years: Connective tissue/rheumatic disease	1.315	1.126	1.536
Charlson comorbidity in previous 5 years: Peptic ulcer disease	0.904	0.806	1.013
Charlson comorbidity in previous 5 years: Mild liver disease	1.254	1.05	1.498
Charlson comorbidity in previous 5 years: Diabetes without complications	0.908	0.829	0.995
Charlson comorbidity in previous 5 years: Diabetes with complications	1.029	0.96	1.102
Charlson comorbidity in previous 5 years: Hemiplegia or paraplegia	1.195	0.99	1.443
Charlson comorbidity in previous 5 years: Renal disease	1.163	1.095	1.235
Charlson comorbidity in previous 5 years: Primary cancer	1.266	1.178	1.36
Charlson comorbidity in previous 5 years: Moderate or severe liver disease	1.712	1.413	2.075
Charlson comorbidity in previous 5 years: Metastatic cancer	1.584	1.372	1.828
Charlson comorbidity in previous 5 years: HIV/AIDS	1.863	0.809	4.288
Year of discharge: 2006 (ref=2005)	1.107	0.998	1.228
Year of discharge: 2007	1.074	0.964	1.196
Year of discharge: 2008	1.005	0.9	1.123
Year of discharge: 2009	1.016	0.907	1.137
Year of discharge: 2010	1.028	0.92	1.149
Year of discharge: 2011	1.023	0.914	1.145
Year of discharge: 2012	0.968	0.864	1.085
Year of discharge: 2013	1.043	0.935	1.163
Year of discharge: 2014	0.42	0.376	0.469
Year of discharge: 2015	1.014	0.911	1.129
Year of discharge: 2016	0.974	0.875	1.085
Year of discharge: 2017	1.089	0.982	1.208
Year of discharge: 2018	0.977	0.878	1.087
Year of discharge: 2019	0.865	0.769	0.973

© 2022 Saxena FE et al. *JAMA Network Open.*

eTable 18. Cox Proportional Hazards Model for All-Cause Readmissions (Death as Competing Risk) Within 90 d Among Individuals With Congestive Heart Failure

Parameter	Hazard Ratio	95% Hazard Ratio Confidence Limits	
Office visit (PC or relevant specialist) within 7 days after discharge	0.975	0.955	0.994
Females: 46 - 64 (ref= females 20 - 45)	0.949	0.825	1.091
Females: 65 - 74	1.022	0.892	1.172
Females: 75 - 84	1.128	0.986	1.29
Females: 85+	1.269	1.109	1.452
Males: 20 - 45	0.797	0.671	0.948
Males: 46 - 64	0.903	0.788	1.035
Males: 65 - 74	1.009	0.881	1.155
Males 75 - 84	1.129	0.987	1.291
Males: 85+	1.269	1.108	1.454
Income quintile: 1 (ref=income quintile 5)	1.095	1.058	1.132
Income quintile: 2	1.039	1.004	1.075
Income quintile: 3	1.021	0.986	1.058
Income quintile: 4	1.007	0.971	1.044
Income quintile: Missing	1.042	0.876	1.24
Urban (RIO < 10) (ref=rural)	1.008	0.982	1.034
RIO (missing)	1.058	0.965	1.161
Ambulatory visit to a comprehensive PC physician in the past year	1.081	1.037	1.126
Usual provider of care: FHT (ref=no usual provider of care)	0.413	0.38	0.448
Usual provider of care: non-FHT (ref=no usual provider of care)	0.408	0.376	0.442
Specialty of discharging physician: Cardiologist (ref=GP/FP)	0.848	0.821	0.876
Specialty of discharging physician: GIM	0.976	0.95	1.003
Specialty of discharing physician: Other	0.965	0.926	1.007
CHF severity conditions: Ischemic heart disease	1.008	0.982	1.034
CHF severity conditions: Other cardiovascular disease	1.097	1.068	1.126
CHF severity conditions: Shock	0.929	0.759	1.138
CHF severity conditions: Peripheral vascular disease	1.129	1.061	1.203
CHF severity conditions: Arrhythmia	0.989	0.967	1.012
CHF severity conditions: Cerebrovascular disease	1.035	0.937	1.143
CHF severity conditions: Hypertension	0.907	0.887	0.929
CHF severity conditions: COPD	1.042	1.01	1.074
CHF severity conditions: Dementia	1.033	0.96	1.111
CHF severity conditions: Acute renal failure	1.246	1.208	1.286
CHF severity conditions: Chronic renal failure	1.169	1.101	1.241
CHF severity conditions: Diabetes	1.017	0.986	1.05
CHF severity conditions: Non-metastatic cancer	1.309	1.236	1.387
CHF severity conditions: Metastatic cancer	1.376	1.226	1.545
CHF severity conditions: Moderate/severe liver disease	1.402	1.172	1.676
History of AMI in previous 5 years	1.023	0.971	1.079
History of CHF or angina in previous 5 years	1.141	1.096	1.188
History of implantable cardiac defibrillator in previous 3 years	1.008	0.965	1.054
History of permanent pacemake in previous 3 years	1.128	0.83	1.534
Charlson comorbidity in previous 5 years: Myocardial infarction	1.063	1.011	1.118
Charlson comorbidity in previous 5 years: CHF	1.197	1.15	1.246
Charlson comorbidity in previous 5 years: Peripheral vascular disease	1.123	1.081	1.167
Charlson comorbidity in previous 5 years: Cerebrovascular disease	1.082	1.04	1.127
Charlson comorbidity in previous 5 years: Dementia	1.096	1.028	1.169
Charlson comorbidity in previous 5 years: COPD	1.256	1.22	1.293
Charlson comorbidity in previous 5 years: Connective tissue/rheumatic disease	1.249	1.154	1.352
Charlson comorbidity in previous 5 years: Peptic ulcer disease	1.076	1.019	1.136
Charlson comorbidity in previous 5 years: Mild liver disease	1.327	1.22	1.444
Charlson comorbidity in previous 5 years: Diabetes without complications	1.057	1.012	1.105
Charlson comorbidity in previous 5 years: Diabetes with complications	1.162	1.122	1.203
Charlson comorbidity in previous 5 years: Hemiplegia or paraplegia	1.047	0.947	1.159
Charlson comorbidity in previous 5 years: Renal disease	1.223	1.185	1.262
Charlson comorbidity in previous 5 years: Primary cancer	1.082	1.039	1.126
Charlson comorbidity in previous 5 years: Moderate or severe liver disease	1.548	1.395	1.718
Charlson comorbidity in previous 5 years: Metastatic cancer	1.179	1.08	1.287
Charlson comorbidity in previous 5 years: HIV/AIDS	1.717	1.096	2.689
Year of discharge: 2006 (ref=2005)	0.981	0.93	1.034
Year of discharge: 2007	0.973	0.921	1.027
Year of discharge: 2008	0.941	0.89	0.994
Year of discharge: 2009	1.013	0.958	1.071
Year of discharge: 2010	1.01	0.955	1.067
Year of discharge: 2011	1.008	0.953	1.066
Year of discharge: 2012	1.013	0.958	1.072
Year of discharge: 2013	1.024	0.969	1.081
Year of discharge: 2014	0.848	0.801	0.898
Year of discharge: 2015	0.992	0.94	1.047
Year of discharge: 2016	0.964	0.913	1.017
Year of discharge: 2017	0.97	0.92	1.023
Year of discharge: 2018	0.941	0.891	0.993
Year of discharge: 2019	0.934	0.881	0.99

© 2022 Saxena FE et al. *JAMA Network Open.*

eTable 19. Cox Proportional Hazards Model for Major Cardiac Event (Death as Competing Risk) Within 90 d Among Individuals With Congestive Heart Failure

Parameter	Hazard Ratio	95% Hazard Ratio Confidence Limits	
Office visit (PC or relevant specialist) within 7 days after discharge	1	0.97	1.031
Females: 46 - 64 (ref= females 20 - 45)	1.236	0.969	1.575
Females: 65 - 74	1.406	1.109	1.784
Females: 75 - 84	1.62	1.281	2.049
Females: 85+	1.951	1.543	2.469
Males: 20 - 45	1.078	0.808	1.439
Males: 46 - 64	1.213	0.956	1.538
Males: 65 - 74	1.496	1.182	1.894
Males 75 - 84	1.611	1.274	2.038
Males: 85+	1.925	1.52	2.438
Income quintile: 1 (ref=income quintile 5)	1.119	1.065	1.176
Income quintile: 2	1.062	1.01	1.117
Income quintile: 3	1.033	0.981	1.089
Income quintile: 4	1	0.947	1.055
Income quintile: Missing	1.078	0.835	1.392
Urban (RIO < 10) (ref=rural)	1.016	0.978	1.055
RIO (missing)	1.042	0.906	1.199
Ambulatory visit to a comprehensive PC physician in the past year	1.105	1.039	1.175
Usual provider of care: FHT (ref=no usual provider of care)	0.45	0.398	0.51
Usual provider of care: non-FHT (ref=no usual provider of care)	0.452	0.4	0.511
Specialty of discharging physician: Cardiologist (ref=GP/FP)	0.878	0.837	0.92
Specialty of discharging physician: GIM	1	0.961	1.04
Specialty of discharing physician: Other	0.841	0.787	0.898
CHF severity conditions: Ischemic heart disease	1.117	1.077	1.159
CHF severity conditions: Other cardiovascular disease	1.141	1.098	1.186
CHF severity conditions: Shock	1.211	0.93	1.575
CHF severity conditions: Peripheral vascular disease	1.052	0.957	1.155
CHF severity conditions: Arrhythmia	0.982	0.95	1.016
CHF severity conditions: Cerebrovascular disease	0.953	0.822	1.105
CHF severity conditions: Hypertension	0.928	0.897	0.961
CHF severity conditions: COPD	0.945	0.902	0.991
CHF severity conditions: Dementia	0.926	0.828	1.036
CHF severity conditions: Acute renal failure	1.282	1.226	1.341
CHF severity conditions: Chronic renal failure	1.128	1.034	1.23
CHF severity conditions: Diabetes	1.091	1.041	1.142
CHF severity conditions: Non-metastatic cancer	1.079	0.983	1.186
CHF severity conditions: Metastatic cancer	1.145	0.937	1.398
CHF severity conditions: Moderate/severe liver disease	0.79	0.553	1.129
History of AMI in previous 5 years	1.138	1.055	1.227
History of CHF or angina in previous 5 years	1.159	1.092	1.23
History of implantable cardiac defibrillator in previous 3 years	1.026	0.964	1.092
History of permanent pacemake in previous 3 years	1.167	0.77	1.77
Charlson comorbidity in previous 5 years: Myocardial infarction	1.092	1.016	1.175
Charlson comorbidity in previous 5 years: CHF	1.329	1.254	1.409
Charlson comorbidity in previous 5 years: Peripheral vascular disease	1.142	1.081	1.207
Charlson comorbidity in previous 5 years: Cerebrovascular disease	1.11	1.048	1.176
Charlson comorbidity in previous 5 years: Dementia	0.943	0.854	1.041
Charlson comorbidity in previous 5 years: COPD	1.113	1.066	1.163
Charlson comorbidity in previous 5 years: Connective tissue/rheumatic disease	0.95	0.835	1.082
Charlson comorbidity in previous 5 years: Peptic ulcer disease	1.006	0.927	1.092
Charlson comorbidity in previous 5 years: Mild liver disease	1.211	1.065	1.377
Charlson comorbidity in previous 5 years: Diabetes without complications	1.061	0.995	1.132
Charlson comorbidity in previous 5 years: Diabetes with complications	1.139	1.082	1.199
Charlson comorbidity in previous 5 years: Hemiplegia or paraplegia	0.949	0.815	1.104
Charlson comorbidity in previous 5 years: Renal disease	1.121	1.071	1.173
Charlson comorbidity in previous 5 years: Primary cancer	0.97	0.912	1.032
Charlson comorbidity in previous 5 years: Moderate or severe liver disease	1.088	0.912	1.298
Charlson comorbidity in previous 5 years: Metastatic cancer	0.924	0.797	1.071
Charlson comorbidity in previous 5 years: HIV/AIDS	2	1.068	3.746
Year of discharge: 2006 (ref=2005)	0.922	0.855	0.994
Year of discharge: 2007	0.91	0.842	0.984
Year of discharge: 2008	0.879	0.812	0.951
Year of discharge: 2009	0.924	0.852	1.001
Year of discharge: 2010	0.937	0.865	1.016
Year of discharge: 2011	0.91	0.839	0.987
Year of discharge: 2012	0.909	0.838	0.986
Year of discharge: 2013	0.972	0.899	1.051
Year of discharge: 2014	0.757	0.697	0.823
Year of discharge: 2015	0.869	0.804	0.94
Year of discharge: 2016	0.87	0.804	0.94
Year of discharge: 2017	0.859	0.795	0.929
Year of discharge: 2018	0.841	0.777	0.909
Year of discharge: 2019	0.815	0.748	0.889

© 2022 Saxena FE et al. *JAMA Network Open.*

eTable 20. Cox Proportional Hazards Model for Death Within 30 d Among Individuals With Chronic Obstructive Pulmonary Disease

Parameter	Hazard Ratio	95% Hazard Ratio Confidence Limits	
Office visit (PC or relevant specialist) within 7 days after discharge	1.044	0.95	1.147
Females: 46 - 64 (ref= females 20 - 45)	1.544	0.624	3.817
Females: 65 - 74	2.607	1.068	6.367
Females: 75 - 84	3.311	1.359	8.067
Females: 85+	4.374	1.783	10.734
Males: 20 - 45	1.196	0.322	4.452
Males: 46 - 64	2.723	1.113	6.662
Males: 65 - 74	3.649	1.5	8.878
Males 75 - 84	3.996	1.644	9.712
Males: 85+	5.884	2.411	14.36
Income quintile: 1 (ref=income quintile 5)	1.116	0.951	1.31
Income quintile: 2	1.134	0.963	1.335
Income quintile: 3	1.089	0.92	1.29
Income quintile: 4	1.087	0.913	1.294
Income quintile: Missing	1.283	0.594	2.77
Urban (RIO < 10) (ref=rural)	0.964	0.858	1.082
RIO (missing)	1.075	0.743	1.553
Ambulatory visit to a comprehensive PC physician in the past year	0.99	0.821	1.195
Usual provider of care: FHT (ref=no usual provider of care)	0.055	0.045	0.067
Usual provider of care: non-FHT (ref=no usual provider of care)	0.057	0.047	0.069
Specialty of discharging physician: GIM	0.963	0.853	1.088
Specialty of discharing physician: Other	1.008	0.837	1.214
Specialty of discharing physician: Respirologist	1.075	0.896	1.29
COPD severity conditions: Ischemic heart disease	0.919	0.78	1.083
COPD severity conditions: Other cardiovascular disease	1.353	1.127	1.625
COPD severity conditions: Shock	0.711	0.231	2.186
COPD severity conditions: Peripheral vascular disease	1.33	0.988	1.79
COPD severity conditions: Arrhythmia	1.058	0.921	1.216
COPD severity conditions: Cerebrovascular disease	1.611	1.055	2.461
COPD severity conditions: Hypertension	0.803	0.712	0.906
COPD severity conditions: Dementia	1.327	0.996	1.768
COPD severity conditions: CHF	1.338	1.171	1.528
History of COPD in previous 5 years	1.049	0.79	1.393
Other chronic respiratory disease	2.201	1.864	2.6
Charlson comorbidity in previous 5 years: Myocardial infarction	1.222	1.048	1.425
Charlson comorbidity in previous 5 years: CHF	1.168	1.016	1.343
Charlson comorbidity in previous 5 years: Peripheral vascular disease	0.999	0.826	1.21
Charlson comorbidity in previous 5 years: Cerebrovascular disease	0.956	0.777	1.177
Charlson comorbidity in previous 5 years: Dementia	1.625	1.242	2.127
Charlson comorbidity in previous 5 years: COPD	1.22	0.921	1.615
Charlson comorbidity in previous 5 years: Connective tissue/rheumatic disease	0.623	0.386	1.007
Charlson comorbidity in previous 5 years: Peptic ulcer disease	0.776	0.57	1.057
Charlson comorbidity in previous 5 years: Mild liver disease	1.089	0.709	1.673
Charlson comorbidity in previous 5 years: Diabetes without complications	0.788	0.648	0.959
Charlson comorbidity in previous 5 years: Diabetes with complications	0.921	0.796	1.066
Charlson comorbidity in previous 5 years: Hemiplegia or paraplegia	1.096	0.669	1.797
Charlson comorbidity in previous 5 years: Renal disease	1.267	1.062	1.51
Charlson comorbidity in previous 5 years: Primary cancer	1.914	1.67	2.193
Charlson comorbidity in previous 5 years: Moderate or severe liver disease	1.235	0.745	2.045
Charlson comorbidity in previous 5 years: Metastatic cancer	5.793	4.896	6.856
Charlson comorbidity in previous 5 years: HIV/AIDS	1.346	0.188	9.658
Year of discharge: 2006 (ref=2005)	0.85	0.676	1.069
Year of discharge: 2007	1.015	0.797	1.293
Year of discharge: 2008	1.011	0.793	1.289
Year of discharge: 2009	1.157	0.908	1.475
Year of discharge: 2010	1.101	0.865	1.401
Year of discharge: 2011	0.981	0.762	1.264
Year of discharge: 2012	1.167	0.916	1.488
Year of discharge: 2013	0.964	0.745	1.248
Year of discharge: 2014	0.409	0.327	0.512
Year of discharge: 2015	1.298	1.028	1.64
Year of discharge: 2016	1.191	0.942	1.505
Year of discharge: 2017	1.139	0.898	1.445
Year of discharge: 2018	1.164	0.913	1.483
Year of discharge: 2019	1.034	0.774	1.381

© 2022 Saxena FE et al. *JAMA Network Open.*

eTable 21. Cox Proportional Hazards Model for All-Cause Readmissions (Death as Competing Risk) Within 30 d Among Individuals With Chronic Obstructive Pulmonary Disease

Parameter	Hazard Ratio	95% Hazard Ratio Confidence Limits	
Office visit (PC or relevant specialist) within 7 days after discharge	0.965	0.929	1.003
Females: 46 - 64 (ref= females 20 - 45)	0.969	0.758	1.239
Females: 65 - 74	1.156	0.906	1.476
Females: 75 - 84	1.21	0.949	1.544
Females: 85+	1.231	0.959	1.58
Males: 20 - 45	0.99	0.695	1.409
Males: 46 - 64	1.168	0.914	1.492
Males: 65 - 74	1.279	1.003	1.631
Males 75 - 84	1.355	1.063	1.727
Males: 85+	1.355	1.057	1.737
Income quintile: 1 (ref=income quintile 5)	1.098	1.029	1.17
Income quintile: 2	1.034	0.968	1.106
Income quintile: 3	1.03	0.961	1.103
Income quintile: 4	1.023	0.953	1.099
Income quintile: Missing	1.037	0.755	1.423
Urban (RIO < 10) (ref=rural)	1.006	0.961	1.053
RIO (missing)	1.096	0.938	1.28
Ambulatory visit to a comprehensive PC physician in the past year	1.04	0.964	1.123
Usual provider of care: FHT (ref=no usual provider of care)	0.314	0.269	0.367
Usual provider of care: non-FHT (ref=no usual provider of care)	0.314	0.269	0.366
Specialty of discharging physician: GIM	1.048	1	1.099
Specialty of discharing physician: Other	0.994	0.921	1.072
Specialty of discharing physician: Respirologist	1.088	1.013	1.169
COPD severity conditions: Ischemic heart disease	1.07	1.005	1.139
COPD severity conditions: Other cardiovascular disease	1.134	1.047	1.227
COPD severity conditions: Shock	1.043	0.653	1.667
COPD severity conditions: Peripheral vascular disease	1.161	1.02	1.321
COPD severity conditions: Arrhythmia	1.157	1.093	1.224
COPD severity conditions: Cerebrovascular disease	0.997	0.816	1.219
COPD severity conditions: Hypertension	0.918	0.876	0.962
COPD severity conditions: Dementia	0.995	0.862	1.149
COPD severity conditions: CHF	1.23	1.164	1.3
History of COPD in previous 5 years	1.229	1.093	1.383
Other chronic respiratory disease	1.4	1.29	1.52
Charlson comorbidity in previous 5 years: Myocardial infarction	1.117	1.05	1.188
Charlson comorbidity in previous 5 years: CHF	1.289	1.22	1.361
Charlson comorbidity in previous 5 years: Peripheral vascular disease	1.064	0.986	1.147
Charlson comorbidity in previous 5 years: Cerebrovascular disease	1.104	1.016	1.199
Charlson comorbidity in previous 5 years: Dementia	1.24	1.095	1.404
Charlson comorbidity in previous 5 years: COPD	1.15	1.023	1.293
Charlson comorbidity in previous 5 years: Connective tissue/rheumatic disease	1.188	1.033	1.367
Charlson comorbidity in previous 5 years: Peptic ulcer disease	1.035	0.926	1.157
Charlson comorbidity in previous 5 years: Mild liver disease	1.289	1.109	1.498
Charlson comorbidity in previous 5 years: Diabetes without complications	1.034	0.964	1.109
Charlson comorbidity in previous 5 years: Diabetes with complications	1.109	1.047	1.175
Charlson comorbidity in previous 5 years: Hemiplegia or paraplegia	1.181	0.979	1.424
Charlson comorbidity in previous 5 years: Renal disease	1.315	1.225	1.412
Charlson comorbidity in previous 5 years: Primary cancer	1.188	1.115	1.265
Charlson comorbidity in previous 5 years: Moderate or severe liver disease	1.382	1.131	1.688
Charlson comorbidity in previous 5 years: Metastatic cancer	1.89	1.702	2.099
Charlson comorbidity in previous 5 years: HIV/AIDS	1.557	0.861	2.817
Year of discharge: 2006 (ref=2005)	0.905	0.83	0.987
Year of discharge: 2007	0.936	0.853	1.026
Year of discharge: 2008	0.923	0.841	1.014
Year of discharge: 2009	0.893	0.81	0.985
Year of discharge: 2010	0.992	0.902	1.09
Year of discharge: 2011	0.891	0.806	0.984
Year of discharge: 2012	1.021	0.929	1.122
Year of discharge: 2013	0.943	0.855	1.04
Year of discharge: 2014	0.8	0.723	0.885
Year of discharge: 2015	0.977	0.888	1.075
Year of discharge: 2016	0.921	0.837	1.014
Year of discharge: 2017	0.897	0.815	0.987
Year of discharge: 2018	0.965	0.876	1.063
Year of discharge: 2019	1.029	0.92	1.149

© 2022 Saxena FE et al. *JAMA Network Open.*

eTable 22. Cox Proportional Hazards Model for Readmission for Chronic Obstructive Pulmonary Disease (COPD) or COPD-Related Condition (Death as Competing Risk) Within 90 d Among Individuals With COPD

Parameter	Hazard Ratio	95% Hazard Ratio Confidence Limits	
Office visit (PC or relevant specialist) within 7 days after discharge	0.943	0.89	1
Females: 46 - 64 (ref= females 20 - 45)	0.982	0.684	1.41
Females: 65 - 74	1.16	0.81	1.662
Females: 75 - 84	1.124	0.785	1.61
Females: 85+	1.028	0.709	1.493
Males: 20 - 45	1.062	0.646	1.748
Males: 46 - 64	1.235	0.861	1.771
Males: 65 - 74	1.384	0.967	1.98
Males 75 - 84	1.364	0.954	1.951
Males: 85+	1.276	0.883	1.843
Income quintile: 1 (ref=income quintile 5)	1.132	1.024	1.25
Income quintile: 2	1.079	0.973	1.197
Income quintile: 3	1.109	0.997	1.233
Income quintile: 4	1.035	0.926	1.157
Income quintile: Missing	1.695	1.149	2.501
Urban (RIO < 10) (ref=rural)	1.075	1.003	1.152
RIO (missing)	1.036	0.821	1.307
Ambulatory visit to a comprehensive PC physician in the past year	1.067	0.949	1.199
Usual provider of care: FHT (ref=no usual provider of care)	0.401	0.312	0.517
Usual provider of care: non-FHT (ref=no usual provider of care)	0.391	0.304	0.501
Specialty of discharging physician: GIM	1.076	1.001	1.155
Specialty of discharing physician: Other	0.857	0.757	0.971
Specialty of discharing physician: Respirologist	1.208	1.09	1.338
COPD severity conditions: Ischemic heart disease	0.936	0.845	1.037
COPD severity conditions: Other cardiovascular disease	0.939	0.821	1.074
COPD severity conditions: Shock	1.02	0.461	2.259
COPD severity conditions: Peripheral vascular disease	0.935	0.747	1.17
COPD severity conditions: Arrhythmia	1.029	0.937	1.129
COPD severity conditions: Cerebrovascular disease	0.973	0.706	1.34
COPD severity conditions: Hypertension	0.932	0.868	1.002
COPD severity conditions: Dementia	0.953	0.752	1.208
COPD severity conditions: CHF	0.961	0.876	1.053
History of COPD in previous 5 years	1.3	1.083	1.561
Other chronic respiratory disease	1.098	0.957	1.261
Charlson comorbidity in previous 5 years: Myocardial infarction	0.988	0.895	1.092
Charlson comorbidity in previous 5 years: CHF	1.183	1.086	1.287
Charlson comorbidity in previous 5 years: Peripheral vascular disease	0.969	0.857	1.096
Charlson comorbidity in previous 5 years: Cerebrovascular disease	1.012	0.884	1.158
Charlson comorbidity in previous 5 years: Dementia	1.035	0.835	1.283
Charlson comorbidity in previous 5 years: COPD	1.472	1.227	1.766
Charlson comorbidity in previous 5 years: Connective tissue/rheumatic disease	1.286	1.039	1.592
Charlson comorbidity in previous 5 years: Peptic ulcer disease	0.925	0.775	1.104
Charlson comorbidity in previous 5 years: Mild liver disease	1.149	0.913	1.446
Charlson comorbidity in previous 5 years: Diabetes without complications	0.996	0.896	1.108
Charlson comorbidity in previous 5 years: Diabetes with complications	0.94	0.855	1.034
Charlson comorbidity in previous 5 years: Hemiplegia or paraplegia	1.207	0.896	1.627
Charlson comorbidity in previous 5 years: Renal disease	1.101	0.977	1.242
Charlson comorbidity in previous 5 years: Primary cancer	1.023	0.925	1.132
Charlson comorbidity in previous 5 years: Moderate or severe liver disease	0.72	0.473	1.096
Charlson comorbidity in previous 5 years: Metastatic cancer	1.182	0.981	1.424
Charlson comorbidity in previous 5 years: HIV/AIDS	1.184	0.472	2.971
Year of discharge: 2006 (ref=2005)	0.771	0.681	0.873
Year of discharge: 2007	0.807	0.705	0.925
Year of discharge: 2008	0.746	0.649	0.858
Year of discharge: 2009	0.731	0.63	0.848
Year of discharge: 2010	0.865	0.753	0.994
Year of discharge: 2011	0.819	0.708	0.947
Year of discharge: 2012	0.792	0.687	0.915
Year of discharge: 2013	0.753	0.649	0.873
Year of discharge: 2014	0.74	0.638	0.858
Year of discharge: 2015	0.803	0.697	0.927
Year of discharge: 2016	0.747	0.649	0.861
Year of discharge: 2017	0.717	0.62	0.829
Year of discharge: 2018	0.812	0.703	0.937
Year of discharge: 2019	0.83	0.702	0.982

© 2022 Saxena FE et al. *JAMA Network Open.*

eTable 23. Cox Proportional Hazards Model for Death Within 90 Days Among Individuals With Chronic Obstructive Pulmonary Disease

Parameter	Hazard Ratio	95% Hazard Ratio Confidence Limits	
Office visit (PC or relevant specialist) within 7 days after discharge	0.956	0.906	1.008
Females: 46 - 64 (ref= females 20 - 45)	2.249	1.234	4.099
Females: 65 - 74	3.414	1.882	6.193
Females: 75 - 84	4.73	2.611	8.571
Females: 85+	5.75	3.161	10.457
Males: 20 - 45	1.473	0.659	3.294
Males: 46 - 64	3.45	1.899	6.267
Males: 65 - 74	4.834	2.669	8.755
Males 75 - 84	5.666	3.13	10.255
Males: 85+	7.58	4.178	13.753
Income quintile: 1 (ref=income quintile 5)	1.101	1.008	1.203
Income quintile: 2	1.068	0.975	1.169
Income quintile: 3	1.026	0.934	1.126
Income quintile: 4	1.02	0.926	1.125
Income quintile: Missing	0.902	0.565	1.441
Urban (RIO < 10) (ref=rural)	0.981	0.92	1.046
RIO (missing)	1.026	0.816	1.291
Ambulatory visit to a comprehensive PC physician in the past year	1.008	0.906	1.121
Usual provider of care: FHT (ref=no usual provider of care)	0.056	0.049	0.065
Usual provider of care: non-FHT (ref=no usual provider of care)	0.058	0.051	0.067
Specialty of discharging physician: GIM	0.969	0.906	1.037
Specialty of discharing physician: Other	1.109	1.004	1.224
Specialty of discharing physician: Respirologist	0.996	0.898	1.105
COPD severity conditions: Ischemic heart disease	0.939	0.859	1.026
COPD severity conditions: Other cardiovascular disease	1.404	1.267	1.557
COPD severity conditions: Shock	0.662	0.318	1.378
COPD severity conditions: Peripheral vascular disease	1.336	1.121	1.592
COPD severity conditions: Arrhythmia	1.042	0.962	1.128
COPD severity conditions: Cerebrovascular disease	1.232	0.943	1.611
COPD severity conditions: Hypertension	0.788	0.736	0.843
COPD severity conditions: Dementia	1.475	1.257	1.731
COPD severity conditions: CHF	1.333	1.238	1.437
History of COPD in previous 5 years	1.067	0.91	1.252
Other chronic respiratory disease	1.844	1.665	2.042
Charlson comorbidity in previous 5 years: Myocardial infarction	1.132	1.039	1.232
Charlson comorbidity in previous 5 years: CHF	1.276	1.183	1.377
Charlson comorbidity in previous 5 years: Peripheral vascular disease	1.021	0.918	1.136
Charlson comorbidity in previous 5 years: Cerebrovascular disease	0.994	0.885	1.116
Charlson comorbidity in previous 5 years: Dementia	1.391	1.188	1.629
Charlson comorbidity in previous 5 years: COPD	1.174	1.002	1.376
Charlson comorbidity in previous 5 years: Connective tissue/rheumatic disease	0.798	0.629	1.012
Charlson comorbidity in previous 5 years: Peptic ulcer disease	1.005	0.859	1.176
Charlson comorbidity in previous 5 years: Mild liver disease	1.268	1.016	1.583
Charlson comorbidity in previous 5 years: Diabetes without complications	0.922	0.83	1.024
Charlson comorbidity in previous 5 years: Diabetes with complications	0.994	0.916	1.077
Charlson comorbidity in previous 5 years: Hemiplegia or paraplegia	1.204	0.928	1.562
Charlson comorbidity in previous 5 years: Renal disease	1.294	1.175	1.424
Charlson comorbidity in previous 5 years: Primary cancer	2.018	1.871	2.178
Charlson comorbidity in previous 5 years: Moderate or severe liver disease	1.63	1.245	2.134
Charlson comorbidity in previous 5 years: Metastatic cancer	5.654	5.121	6.242
Charlson comorbidity in previous 5 years: HIV/AIDS	0.564	0.115	2.756
Year of discharge: 2006 (ref=2005)	0.909	0.802	1.03
Year of discharge: 2007	1.01	0.884	1.153
Year of discharge: 2008	0.972	0.849	1.112
Year of discharge: 2009	1.028	0.896	1.178
Year of discharge: 2010	1.108	0.971	1.265
Year of discharge: 2011	1.038	0.903	1.193
Year of discharge: 2012	1.131	0.988	1.293
Year of discharge: 2013	1.038	0.903	1.194
Year of discharge: 2014	0.413	0.359	0.476
Year of discharge: 2015	1.194	1.048	1.362
Year of discharge: 2016	1.093	0.957	1.248
Year of discharge: 2017	1.077	0.944	1.23
Year of discharge: 2018	0.948	0.821	1.094
Year of discharge: 2019	0.947	0.803	1.115

© 2022 Saxena FE et al. *JAMA Network Open.*

eTable 24. Cox Proportional Hazards Model for All-Cause Readmission (Death as Competing Risk) Within 90 d Among Individuals With Chronic Obstructive Pulmonary Disease

Parameter	Hazard Ratio	95% Hazard Ratio Confidence Limits	
Office visit (PC or relevant specialist) within 7 days after discharge	0.951	0.927	0.976
Females: 46 - 64 (ref= females 20 - 45)	1.002	0.849	1.182
Females: 65 - 74	1.152	0.978	1.358
Females: 75 - 84	1.259	1.069	1.483
Females: 85+	1.31	1.107	1.55
Males: 20 - 45	1.045	0.826	1.322
Males: 46 - 64	1.18	1	1.391
Males: 65 - 74	1.296	1.1	1.527
Males 75 - 84	1.37	1.164	1.613
Males: 85+	1.478	1.251	1.746
Income quintile: 1 (ref=income quintile 5)	1.093	1.047	1.142
Income quintile: 2	1.042	0.996	1.089
Income quintile: 3	1.015	0.969	1.063
Income quintile: 4	1.01	0.962	1.059
Income quintile: Missing	1.045	0.851	1.284
Urban (RIO < 10) (ref=rural)	1.02	0.989	1.052
RIO (missing)	1.154	1.04	1.28
Ambulatory visit to a comprehensive PC physician in the past year	1.084	1.029	1.142
Usual provider of care: FHT (ref=no usual provider of care)	0.325	0.29	0.364
Usual provider of care: non-FHT (ref=no usual provider of care)	0.325	0.291	0.364
Specialty of discharging physician: GIM	1.018	0.986	1.051
Specialty of discharing physician: Other	0.977	0.928	1.029
Specialty of discharing physician: Respirologist	1.03	0.981	1.082
COPD severity conditions: Ischemic heart disease	1.028	0.984	1.074
COPD severity conditions: Other cardiovascular disease	1.122	1.061	1.186
COPD severity conditions: Shock	1.015	0.732	1.408
COPD severity conditions: Peripheral vascular disease	1.134	1.035	1.243
COPD severity conditions: Arrhythmia	1.131	1.088	1.176
COPD severity conditions: Cerebrovascular disease	1.03	0.897	1.182
COPD severity conditions: Hypertension	0.915	0.886	0.944
COPD severity conditions: Dementia	1.09	0.991	1.198
COPD severity conditions: CHF	1.214	1.169	1.261
History of COPD in previous 5 years	1.226	1.131	1.33
Other chronic respiratory disease	1.337	1.262	1.417
Charlson comorbidity in previous 5 years: Myocardial infarction	1.103	1.057	1.151
Charlson comorbidity in previous 5 years: CHF	1.308	1.26	1.357
Charlson comorbidity in previous 5 years: Peripheral vascular disease	1.095	1.041	1.153
Charlson comorbidity in previous 5 years: Cerebrovascular disease	1.119	1.057	1.183
Charlson comorbidity in previous 5 years: Dementia	1.166	1.069	1.272
Charlson comorbidity in previous 5 years: COPD	1.194	1.102	1.295
Charlson comorbidity in previous 5 years: Connective tissue/rheumatic disease	1.164	1.056	1.282
Charlson comorbidity in previous 5 years: Peptic ulcer disease	1.057	0.98	1.139
Charlson comorbidity in previous 5 years: Mild liver disease	1.264	1.139	1.402
Charlson comorbidity in previous 5 years: Diabetes without complications	1.053	1.004	1.104
Charlson comorbidity in previous 5 years: Diabetes with complications	1.165	1.12	1.211
Charlson comorbidity in previous 5 years: Hemiplegia or paraplegia	1.156	1.014	1.319
Charlson comorbidity in previous 5 years: Renal disease	1.242	1.182	1.305
Charlson comorbidity in previous 5 years: Primary cancer	1.265	1.212	1.32
Charlson comorbidity in previous 5 years: Moderate or severe liver disease	1.432	1.245	1.648
Charlson comorbidity in previous 5 years: Metastatic cancer	2.004	1.864	2.155
Charlson comorbidity in previous 5 years: HIV/AIDS	1.561	1.051	2.318
Year of discharge: 2006 (ref=2005)	0.884	0.834	0.937
Year of discharge: 2007	0.909	0.854	0.968
Year of discharge: 2008	0.897	0.842	0.956
Year of discharge: 2009	0.872	0.817	0.931
Year of discharge: 2010	0.954	0.895	1.017
Year of discharge: 2011	0.891	0.833	0.952
Year of discharge: 2012	0.948	0.888	1.012
Year of discharge: 2013	0.908	0.85	0.971
Year of discharge: 2014	0.812	0.759	0.868
Year of discharge: 2015	0.932	0.874	0.995
Year of discharge: 2016	0.906	0.849	0.966
Year of discharge: 2017	0.85	0.797	0.908
Year of discharge: 2018	0.9	0.842	0.962
Year of discharge: 2019	0.964	0.893	1.04

eTable 25. Cox Proportional Hazards Model for Readmission for Chronic Obstructive Pulmonary Disease (COPD) or COPD-Related Conditions (Death as Competing Risk) Within 90 d Among Individuals With COPD

Parameter	Hazard Ratio	95% Hazard Ratio Confidence Limits	
Office visit (PC or relevant specialist) within 7 days after discharge	0.928	0.893	0.964
Females: 46 - 64 (ref= females 20 - 45)	1.142	0.895	1.456
Females: 65 - 74	1.33	1.044	1.693
Females: 75 - 84	1.319	1.036	1.68
Females: 85+	1.221	0.951	1.568
Males: 20 - 45	1.102	0.786	1.546
Males: 46 - 64	1.379	1.082	1.758
Males: 65 - 74	1.574	1.236	2.003
Males 75 - 84	1.558	1.225	1.983
Males: 85+	1.509	1.179	1.932
Income quintile: 1 (ref=income quintile 5)	1.135	1.063	1.211
Income quintile: 2	1.117	1.045	1.194
Income quintile: 3	1.065	0.993	1.141
Income quintile: 4	1.06	0.987	1.139
Income quintile: Missing	1.546	1.185	2.017
Urban (RIO < 10) (ref=rural)	1.065	1.018	1.115
RIO (missing)	1.028	0.882	1.198
Ambulatory visit to a comprehensive PC physician in the past year	1.09	1.01	1.177
Usual provider of care: FHT (ref=no usual provider of care)	0.393	0.33	0.468
Usual provider of care: non-FHT (ref=no usual provider of care)	0.396	0.333	0.471
Specialty of discharging physician: GIM	1.037	0.99	1.087
Specialty of discharing physician: Other	0.853	0.787	0.925
Specialty of discharing physician: Respirologist	1.153	1.078	1.233
COPD severity conditions: Ischemic heart disease	0.917	0.857	0.981
COPD severity conditions: Other cardiovascular disease	0.981	0.899	1.071
COPD severity conditions: Shock	1.078	0.668	1.74
COPD severity conditions: Peripheral vascular disease	0.936	0.809	1.083
COPD severity conditions: Arrhythmia	0.993	0.934	1.055
COPD severity conditions: Cerebrovascular disease	0.991	0.802	1.223
COPD severity conditions: Hypertension	0.93	0.887	0.975
COPD severity conditions: Dementia	0.977	0.839	1.138
COPD severity conditions: CHF	0.99	0.932	1.051
History of COPD in previous 5 years	1.32	1.171	1.487
Other chronic respiratory disease	1.086	0.991	1.19
Charlson comorbidity in previous 5 years: Myocardial infarction	0.988	0.925	1.055
Charlson comorbidity in previous 5 years: CHF	1.127	1.066	1.192
Charlson comorbidity in previous 5 years: Peripheral vascular disease	1.001	0.925	1.083
Charlson comorbidity in previous 5 years: Cerebrovascular disease	1.048	0.962	1.143
Charlson comorbidity in previous 5 years: Dementia	1.034	0.899	1.189
Charlson comorbidity in previous 5 years: COPD	1.489	1.322	1.676
Charlson comorbidity in previous 5 years: Connective tissue/rheumatic disease	1.109	0.957	1.286
Charlson comorbidity in previous 5 years: Peptic ulcer disease	1.024	0.914	1.147
Charlson comorbidity in previous 5 years: Mild liver disease	1.057	0.902	1.238
Charlson comorbidity in previous 5 years: Diabetes without complications	0.992	0.926	1.062
Charlson comorbidity in previous 5 years: Diabetes with complications	0.96	0.902	1.021
Charlson comorbidity in previous 5 years: Hemiplegia or paraplegia	1.051	0.857	1.29
Charlson comorbidity in previous 5 years: Renal disease	1.04	0.959	1.127
Charlson comorbidity in previous 5 years: Primary cancer	1.066	0.998	1.138
Charlson comorbidity in previous 5 years: Moderate or severe liver disease	0.755	0.578	0.985
Charlson comorbidity in previous 5 years: Metastatic cancer	1.298	1.149	1.466
Charlson comorbidity in previous 5 years: HIV/AIDS	1.18	0.661	2.109
Year of discharge: 2006 (ref=2005)	0.793	0.731	0.86
Year of discharge: 2007	0.807	0.739	0.882
Year of discharge: 2008	0.778	0.71	0.852
Year of discharge: 2009	0.751	0.683	0.826
Year of discharge: 2010	0.862	0.788	0.945
Year of discharge: 2011	0.82	0.746	0.902
Year of discharge: 2012	0.798	0.726	0.876
Year of discharge: 2013	0.782	0.71	0.862
Year of discharge: 2014	0.74	0.673	0.815
Year of discharge: 2015	0.778	0.708	0.855
Year of discharge: 2016	0.788	0.718	0.865
Year of discharge: 2017	0.713	0.649	0.784
Year of discharge: 2018	0.815	0.742	0.895
Year of discharge: 2019	0.824	0.738	0.92

© 2022 Saxena FE et al. *JAMA Network Open.*

eTable 26. Adjusted Hazard Ratios With 95% CIs of Adverse Events for Patients Who Received vs Did Not Receive Early Physician Follow-up, Overall, by Sex, Length of Stay Group, and Subgroup at Increased Risk

Cohort Adverse Outcomes	Adjusted hazard ratio (95% confidence interval)									
	Original cohort	Age ≥ 75 years	Female	Male	LOS 2 days	LOS 3-7 days	LOS >7 days	Any comor-bidity	Low SES	Rural resi-dence
**Acute Myocardial Infarction (AMI), N**										
Unplanned readmission within 30 days of discharge, %	0.99 (0.96, 1.03)	0.97 (0.92, 1.02)	0.97 (0.92, 1.03)	0.99 (0.95, 1.04)	0.92 (0.82, 1.03)	1.03 (0.99, 1.08)	0.96 (0.90, 1.01)	0.97 (0.94, 1.01)	0.96 (0.92, 1.01)	0.99 (0.94, 1.05)
Major cardiac event within 30 days of discharge, %	1.02 (0.97, 1.08)	1.03 (0.95, 1.11)	1.01 (0.92, 1.11)	1.03 (0.96, 1.11)	0.85 (0.69, 1.05)	1.07 (0.99, 1.15)	1.00 (0.91, 1.15)	1.01 (0.96, 1.08)	0.99 (0.92, 1.06)	1.02 (0.93, 1.12)
Death within 30 days of discharge, %	0.92 (0.83, 1.03)	1.04 (0.90, 1.19)	1.02 (0.86, 1.22)	0.88 (0.76, 1.01)	0.99 (0.64, 1.53)	0.87 (0.74, 1.02)	1.02 (0.87, 1.20)	0.93 (0.83, 1.04)	0.89 (0.78, 1.02)	0.91 (0.76, 1.09)
Unplanned readmission within 90 days of discharge, %	0.99 (0.97, 1.02)	1.00 (0.97, 1.04)	0.97 (0.93, 1.01)	1.01 (0.98, 1.04)	0.96 (0.88, 1.04)	1.03 (1.00, 1.07)	0.96 (0.93, 1.00)	0.99 (0.96, 1.01)	0.98 (0.95, 1.01)	0.98 (0.94, 1.02)
Major cardiac event within 90 days of discharge, %	1.02 (0.98, 1.06)	1.03 (0.98, 1.09)	0.98 (0.92, 1.05)	1.04 (0.99, 1.10)	0.93 (0.81, 1.07)	1.05 (1.00, 1.12)	1.00 (0.94, 1.07)	1.01 (0.97, 1.05)	1.01 (0.96, 1.06)	1.02 (0.96, 1.09)
Death within 90 days of discharge, %	0.95 (0.89, 1.01)	0.97 (0.90, 1.05)	0.97 (0.88, 1.07)	0.93 (0.86, 1.01)	1.09 (0.84, 1.42)	0.95 (0.87, 1.04)	0.96 (0.88, 1.05)	0.94 (0.89, 1.01)	0.98 (0.90, 1.05)	0.93 (0.84, 1.03)
**Congestive Heart Failure (CHF)**										
Unplanned readmission within 30 days of discharge, %	1.01 (0.98, 1.04)	1.01 (0.97, 1.05)	1.05 (1.01, 1.10)	0.97 (0.93, 1.01)	1.02 (0.92, 1.13)	1.03 (0.98, 1.07)	1.00 (0.95, 1.05)	1.01 (0.97, 1.04)	1.00 (0.97, 1.04)	1.01 (0.96, 1.07)
Major cardiac event within 30 days of discharge, %	1.02 (0.98, 1.07)	1.02 (0.96, 1.08)	1.05 (0.99, 1.13)	0.99 (0.93, 1.06)	1.10 (0.96, 1.27)	1.05 (0.99, 1.12)	0.97 (0.90, 1.05)	1.02 (0.97, 1.07)	1.00 (0.95, 1.06)	1.06 (0.97, 1.15)
Death within 30 days of discharge, %	0.97 (0.90, 1.05)	1.00 (0.91, 1.09)	1.02 (0.91, 1.14)	0.94 (0.85, 1.04)	0.98 (0.73, 1.31)	0.95 (0.85, 1.07)	1.04 (0.93, 1.15)	0.97 (0.90, 1.05)	1.00 (0.91, 1.09)	0.96 (0.84, 1.09)
Unplanned readmission within 90 days of discharge, %	0.98 (0.96, 0.99)	0.97 (0.94, 0.99)	0.99 (0.96, 1.02)	0.96 (0.93, 0.99)	0.97 (0.91, 1.04)	1.01 (0.98, 1.04)	0.95 (0.92, 0.98)	0.98 (0.96, 0.99)	0.97 (0.95, 0.996)	0.99 (0.95, 1.03)

© 2022 Saxena FE et al. *JAMA Network Open.*

Major cardiac event within 90 days of discharge, %	1.00 (0.97,1.03)	0.98 (0.94, 1.02)	1.01 (0.97, 1.06)	0.99 (0.95, 1.03)	1.06 (0.96, 1.16)	1.04 (0.99, 1.08)	0.95 (0.91, 1.00)	1.00 (0.97, 1.03)	0.99 (0.95, 1.02)	1.02 (0.96, 1.07)
Death within 90 days of discharge, %	0.93 (0.90, 0.97)	0.94 (0.90, 0.98)	0.96 (0.90, 1.02)	0.91 (0.86, 0.96)	1.04 (0.90, 1.21)	0.96 (0.90, 1.02)	0.93 (0.87, 0.98)	0.93 (0.90, 0.97)	0.95 (0.90, 0.998)	0.89 (0.83, 0.95)
**Chronic Obstructive Pulmonary Disease (COPD)**										
Unplanned readmission within 30 days of discharge, %	0.97 (0.93, 1.00)	0.97 (0.92, 1.03)	0.96 (0.91, 1.02)	0.97 (0.92, 1.02)	0.92 (0.82, 1.02)	0.99 (0.94, 1.05)	0.96 (0.90, 1.02)	0.96 (0.93, 1.00)	0.94 (0.90, 0.99)	0.99 (0.94, 1.06)
Readmission for COPD or COPD-related condition within 30 days of discharge, %	0.94 (0.89, 1.00)	0.95 (0.87, 1.03)	0.95 (0.87, 1.03)	0.94 (0.87, 1.02)	0.91 (0.77, 1.06)	0.97 (0.89, 1.05)	0.93 (0.83, 1.03)	0.94 (0.88, 0.995)	0.92 (0.86, 0.99)	0.97 (0.88, 1.06)
Death within 30 days of discharge, %	1.05 (0.95, 1.15)	1.05 (0.93, 1.18)	1.11 (0.96, 1.30)	1.01 (0.90, 1.14)	1.05 (0.76, 1.45)	1.02 (0.89, 1.17)	1.12 (0.97, 1.28)	1.05 (0.95, 1.16)	1.06 (0.94, 1.18)	1.06 (0.92, 1.23)
Unplanned readmission within 90 days of discharge, %	0.95 (0.93, 0.98)	0.96 (0.92, 0.99)	0.97 (0.93, 1.01)	0.94 (0.91, 0.97)	0.94 (0.87, 1.01)	0.97 (0.93, 1.00)	0.95 (0.91, 0.995)	0.95 (0.92, 0.98)	0.94 (0.91, 0.97)	0.96 (0.92, 1.001)
Readmission for COPD or COPD-related condition within 90 days of discharge, %	0.93 (0.89, 0.96)	0.94 (0.89, 0.99)	0.95 (0.90, 1.01)	0.91 (0.86, 0.96)	0.88 (0.79, 0.98)	0.95 (0.90, 1.00)	0.92 (0.86, 0.99)	0.92 (0.89, 0.96)	0.92 (0.88, 0.96)	0.94 (0.88, 0.997)
Death within 90 days of discharge, %	0.96 (0.91, 1.01)	0.95 (0.89, 1.02)	1.07 (0.98, 1.16)	0.90 (0.84, 0.96)	1.01 (0.85, 1.21)	0.93 (0.86, 1.01)	1.01 (0.93, 1.10)	0.95 (0.90, 1.00)	0.95 (0.89, 1.01)	0.97 (0.89, 1.05)

© 2022 Saxena FE et al. *JAMA Network Open.*
